# Therapeutic potential of a TrkB agonistic antibody for Alzheimer's disease

**DOI:** 10.7150/thno.44165

**Published:** 2020-05-23

**Authors:** Shudan Wang, Hongyang Yao, Yihua Xu, Rui Hao, Wen Zhang, Hang Liu, Ying Huang, Wei Guo, Bai Lu

**Affiliations:** 1 School of Pharmaceutical Sciences, IDG/McGovern Institute for Brain Research, Tsinghua University, Beijing, China, 100084.; 2 Beijing Tiantan Hospital, Advanced Innovation Center for Human Brain Protection, Capital Medical University, Beijing, China, 100070.; 3 R&D Center for the Diagnosis and Treatment of Major Brain Diseases, Research Institute of Tsinghua University in Shenzhen, Shenzhen, Guangdong, China, 518057.; 4 Center of Translational Medicine, Tongji Hospital, Tongji University School of Medicine, Shanghai, China, 200065.

**Keywords:** Neurodegeneration, Therapy, Cognition, Antibody drug, Synaptic plasticity

## Abstract

Repeated failures of “Aβ-lowering” therapies call for new targets and therapeutic approaches for Alzheimer's disease (AD). We propose to treat AD by halting neuronal death and repairing synapses using a BDNF-based therapy. To overcome the poor druggability of BDNF, we have developed an agonistic antibody AS86 to mimic the function of BDNF, and evaluate its therapeutic potential for AD.

**Method:** Biochemical, electrophysiological and behavioral techniques were used to investigate the effects of AS86 *in vitro* and *in vivo*.

**Results:** AS86 specifically activated the BDNF receptor TrkB and its downstream signaling, without affecting its other receptor p75^NTR^. It promoted neurite outgrowth, enhanced spine growth and prevented Aβ-induced cell death in cultured neurons, and facilitated Long-Term Potentiation (LTP) in hippocampal slices. A single-dose tail-vein injection of AS86 activated TrkB signaling in the brain, with a half-life of 6 days in the blood and brain. Bi-weekly peripheral administration of AS86 rescued the deficits in object-recognition memory in the APP/PS1 mouse model. AS86 also reversed spatial memory deficits in the 11-month, but not 14-month old AD mouse model.

**Conclusion:** These results demonstrate the potential of AS86 in AD therapy, suggesting that neuronal and/or synaptic repair as an alternative therapeutic strategy for AD.

## Introduction

Great progress has been made in elucidating pathogenic mechanisms of neurodegenerative diseases such as Alzheimer's disease (AD). There is a general understanding that the accumulation of toxic protein aggregates such as Amyloid β (Aβ) and tau in the brain is the major pathogenic factor for AD and perhaps even the cause of the disease 1-3. Molecular pathways for the pathogenesis of AD have begun to emerge, thanks to the advances in Genome-wide association (GWA) analyses and studies using animal models 4,5. However, the progress in understanding AD biology has not been translated into effective therapy. Hundreds of clinical trials have been conducted, but until now, there are no disease-modifying agents that could halt or slow down AD progression 6,7. Several recent clinical studies have shown that reducing the pathogenic toxins such as Aβ, either by immunological approaches (vaccine, antibodies) or by inhibiting its production (inhibitors for β- or γ-secretase), reduced Aβ pathology but failed to improve cognitive deficits 8. These findings cast doubt on the “toxin-reducing” approach and call for a “paradigm shift” in developing therapies for this dreadful disease.

“Disease-modifying” is defined as a “pharmacologic treatment that retards the underlying process of AD by intervening in the neurobiological processes that constitute the pathology and pathophysiology of the disease and lead to cell death or dysfunction.” 9. One emerging concept is to focus on pathophysiology rather than pathogenesis in developing therapeutic treatment for AD 10. Major pathophysiological hallmarks for AD include neuronal death and synapse degeneration. The progression in AD is reflected by early synaptic deficiency 11-13, followed by progressive neuronal loss 14-17. Increasing evidence suggests that synapse loss or neuronal death, but not toxin accumulation, is highly correlated with the progression of AD 2,7. Synaptic integrity and functions are critical for the maintenance of neural circuits and behavioral functions. It is suggested that there is a ~10% neuronal loss, but perhaps more meaningful is ~20% synapse loss in Mild Cognitive Impairment (MCI). Similarly, synaptic loss (~60%) probably contributed more significantly than neuronal loss (~45%) to the disease manifestation in mild AD 12. Unlike neuronal loss, dysfunctional synapses can be repaired or regenerated because synapses are highly dynamic and plastic 18-20. Therefore, targeting mechanisms that can either stabilize/protect or repair/regenerate synapses would allow treatment of the diseases at a later stage (e.g. in prodromal AD or even mild AD).

BDNF, a member of the neurotrophin family, is by far the best known “synaptogenic” molecule and perhaps the only one proven to facilitate synaptic function in humans 21-23. The biological functions of BDNF are mediated primarily by its high-affinity receptor TrkB (Tropomyosin Receptor Kinases B), which activates three major downstream signaling pathways: phosphatidylinositol-3 kinase (PI3 kinase)/Akt; Ras/extracellular regulated kinase (Erk); and PLCγ/PKC 24. Numerous studies have demonstrated that BDNF potentiates synaptic transmission, modulates synaptic plasticity, and induces synapse formation 25-28, both during development and in the adult 23. Behavioral experiments have revealed a role of BDNF in the formation and retention of hippocampal-dependent memory, fear memory extinction, motor learning, as well as mood control 29,30.

Two lines of evidence suggest that the BDNF-TrkB signaling pathway may be a therapeutic target for AD treatment. First, in human subjects, alteration of BDNF expression has been correlated with AD pathophysiology. BDNF mRNA and proteins are decreased in hippocampus, temporal cortex and parietal cortex in the post-mortem brains of human AD subjects 31-33. Moreover, the BDNF Val66Met polymorphism, implicated in a slight reduction in activity-dependent BDNF release 34, 5, has been associated with impairments in episodic memory and hippocampal activity as measured by FDG-PET (Flurodeoxyglucose-Positron emission tomography) in both sporadic and autosomal dominant AD 36-39. Longitudinal studies have revealed an accelerated decline of hippocampal volume and episodic memory, two key endophenotypes, in MCI patients 40-43. Second, extensive studies have documented that BDNF enhances synaptic growth and functions in normal animals 26-28. Moreover, viral expression of BDNF gene in the brain of APP (Amyloid precursor protein) or Tau transgenic AD mice attenuated synapse loss and neuronal abnormality, and rescued the deficits in hippocampal-dependent memory, but did not alleviate Aβ and Tau pathology 44-46. Infusion of BDNF protein into the hippocampus of rat AD model (ventricular injection of Aβ), or daily i.p. injection of a BDNF-mimicking peptide was able to halt neuronal death and ameliorated cognitive impairment 47-49. Therefore, the BDNF-TrkB signaling pathway may be used as a drug target for AD treatment.

Despite its promise, development of drugs targeting the BDNF-TrkB signaling pathway has been challenging. Previous studies have demonstrated that BDNF protein itself may not be used as a therapeutic agent due to its intrinsic physicochemical properties. A number of issues have been raised. First, the half-life of BDNF is very short in both blood (1-10 min) and CSF (1 hour) 50-52. Second, BDNF protein is highly charged and difficult to diffuse in brain or spinal cord parenchyma 53-59. Finally, in addition to activating TrkB, BDNF may also activate its low affinity receptor p75^NTR^, leading to cell death and synaptic depression 60, 61. Efforts have been made to develop a small molecule activator for TrkB 62-66. Unfortunately, more rigorous studies recently demonstrated that these molecules neither bind nor activate TrkB 67,68. To overcome the aforementioned problems, we have attempted to use TrkB agonistic antibodies to activate BDNF signaling. A new TrkB agonist antibody AS86 has been developed, using hybridoma technologies. We evaluated its pharmacological and biological properties, examined its treatment efficacy in both *in vitro* cell model and in an AD mouse model, and outlined the mechanism involved in pathology and synaptic function in AD treatment. Our findings suggest that agonistic antibodies may be a new modality to activate the BDNF-TrkB signaling pathway, paving the way for a new avenue for AD treatment.

## Results

### Characterization of TrkB agonistic antibody AS86

**Figure 1:**
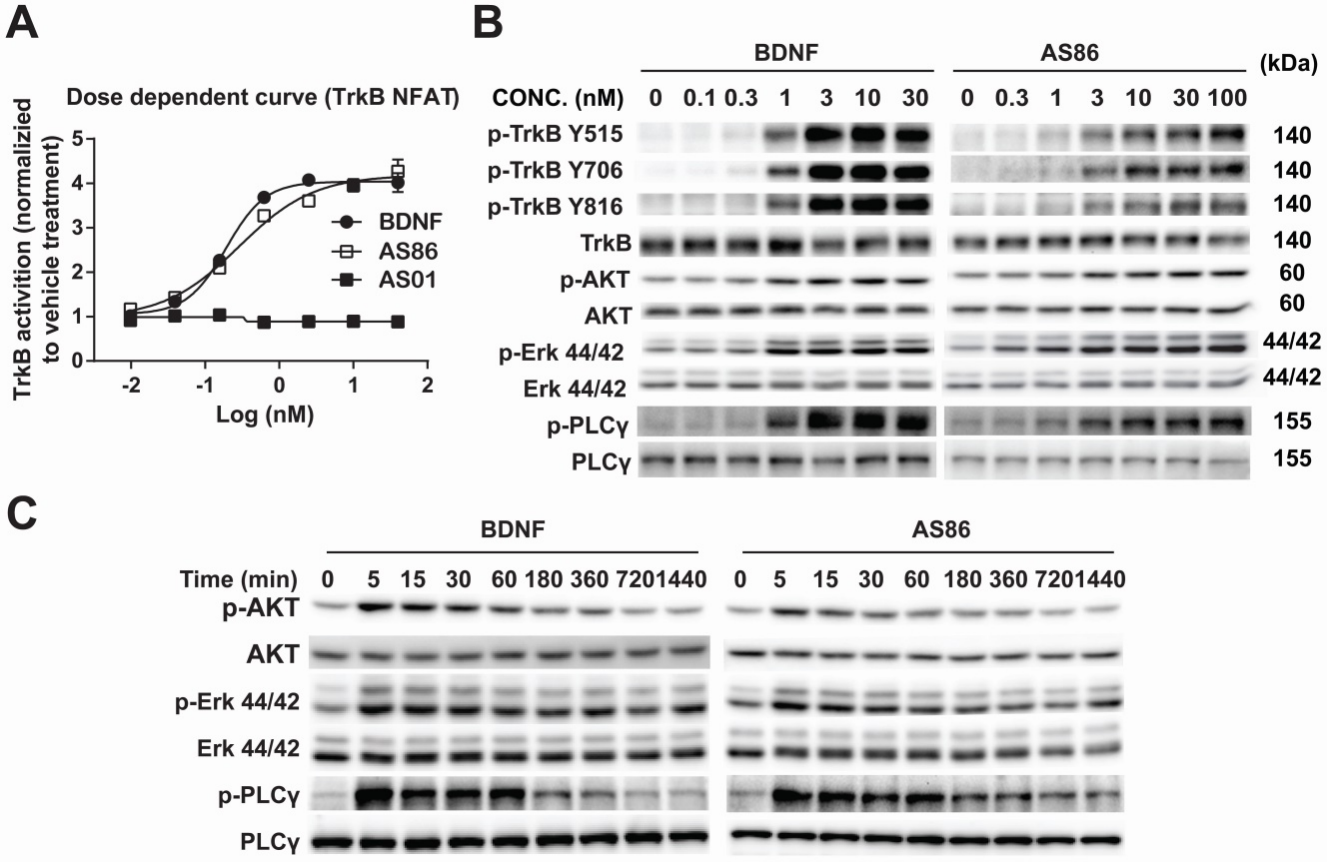
** Potency and signaling of TrkB agonistic antibody AS86.** (**A**) Dose response of TrkB activation by AS86. hTrkB-CHO cells were treated with different doses of TrkB antibodies or BDNF for 4 h, and TrkB activation was analyzed using NFAT assay. (**B**) Dose response of TrkB activation and its downstream signaling in cultured hippocampal neurons. Primary hippocampal neurons (DIV10) were treated with different concentrations of AS86 or BDNF for 30 min, and then the cell lysates were analyzed using Western blotting (N = 3 independent culture experiments, n = 3 repeats for each experiment). Three different tyrosine-phosphorylated sites and downstream signaling pathways were examined. Representative Western blots are presented. (**C**) Time course of AS86 downstream signaling in cultured hippocampal neurons. Primary hippocampal neurons (DIV10) were stimulated with AS86 or BDNF for 0, 5 min, 15 min, 30 min, 60 min, 180 min, 360 min, 720 min and 1440 min, and then the cell lysates were examined for the activation of Akt, Erk and PLCγ with Western blots (N = 3 independent culture experiments, n = 3 repeats for each experiment). Representative Western blots are presented.

Using human TrkB extracellular domain (TrkB-ECD) protein as the antigen, TrkB agonist antibodies were developed by immunization-hybridoma technologies. We have identified a new TrkB monocloncal antibody, AS86, which exhibited a dose-dependent activation of TrkB similar to BDNF, as measured by NFAT assay in TrkB-NFAT-bla CHO-K1 cells (Figure 1A). As a tyrosine receptor kinase (RTK), TrkB activation is mediated by ligand-induced receptor dimerization and endocytosis 24. To determine whether AS86 could induce TrkB endocytosis, we incubated hippocampal neurons with AS86 or BDNF at 37 °C for different amounts of time to allow ligand-induced receptor endocytosis. A biotinylation experiment was performed to detect cell surface TrkB levels. We found that treatment with AS86 elicited a significant decrease in cell surface TrkB as well as total and phosphorylated TrkB, suggesting TrkB endocytosis and degradation upon AS86 binding (Figure S1).

**Figure 2:**
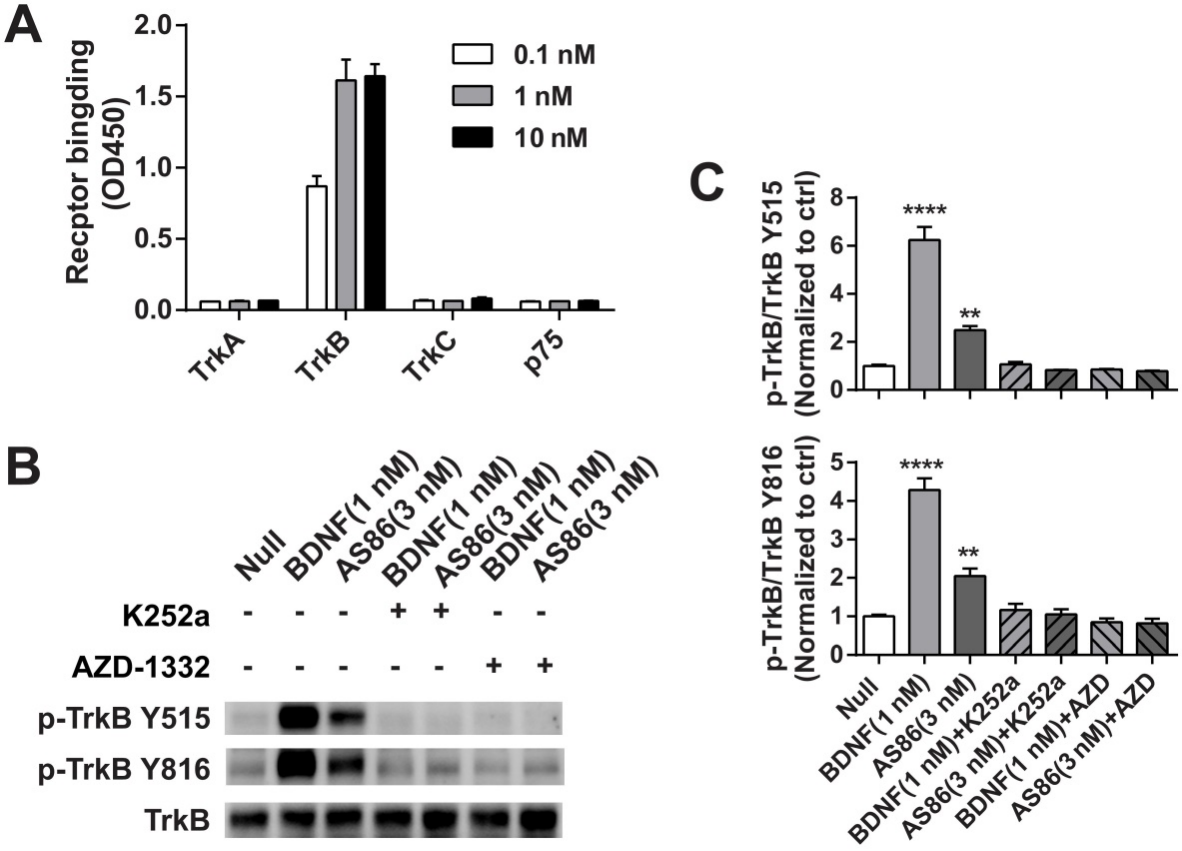
The specificity of TrkB agonistic antibody AS86. (A) AS86 at different concentrations (0.1 nM, 1 nM and 10 nM) was added to the plates coated with different proteins (0.1 μg TrkA, TrkB, TrkC, or p75 respectively), and ELISA was used to examine the binding capacity of AS86. (B, C) Cultured hippocampal neurons (DIV10) were pretreated with the Trk inhibitors k252a (300 nM) or AZD-1332 (100 nM) for 60 min before incubation with mIgG (3 nM), BDNF (1 nM), or AS86 (3 nM) for 15 min (N = 2, n = 3). The Western blots of TrkB Y515 and Y816 sites activation (B) and the quantitative plots (C) are presented. Unless specifically indicated otherwise, statistical analyses in this and all other figures were carried out using one-way ANOVA followed by post hoc test. Symbols for P values (for both ANOVA and Student's *t*-test): *: p < 0.05; **: p < 0.01; ***: p < 0.001; ****: P < 0.0001. The quantification data in this and all other figures were presented as mean ± SEM.

Next, we examined whether AS86 could activate TrkB and its downstream signaling pathways. In cultured hippocampal neurons, AS86 induced TrkB phosphorylation as well as the three major downstream signaling pathways (Akt, Erk and PLCγ) at a concentration as low as 3 nM (Figures 1B and S2A-B). The kinetics of Akt, Erk, and PLCγ signaling by AS86 (10 nM) and BDNF (3 nM) were similar, with the maximal activation at 5 min (Figures 1C and S2C). The antibody binds specifically to TrkB, but not to other neurotrophin receptors such as TrkA, TrkC or p75^NTR^ (Figure 2A), and its ability to induce TrkB tyrosine phosphorylation (Y515 or Y816) was completely blocked by the Trk inhibitors K252a and AZD-1332 (Figure 2B-C). To further demonstrate the specificity of AS86, we performed immunostaining under non-permeable conditions. We found that in cells incubated with AS86, staining with a FITC labeled anti-mouse IgG antibody detected bright TrkB staining in TrkB-CHO or TrkB-PC12 cells, but no signal at all in control TrkA-CHO or normal PC12 cells (Figure S3A), suggesting that AS86 does not bind any other membrane proteins.

To determine which sub-domain of TrkB that AS86 binds to, we constructed a series of truncated TrkB plasmids and transfected them into 293-T cell line. Immunoprecipitation experiments revealed that only the deletion of D5, but not other sub-domains completely eliminated the AS86 binding to TrkB (data not shown). Competition experiments with BDNF at the saturated concentration for TrkB activation (4 nM) and increasing concentrations of AS86 revealed no significant difference between “BDNF alone” and “BDNF plus AS86” (Figure S3B-C), suggesting that AS86 is a non-competitive TrkB agonist.

### Attenuation of Aβ-induced cell death by AS86

**Figure 3:**
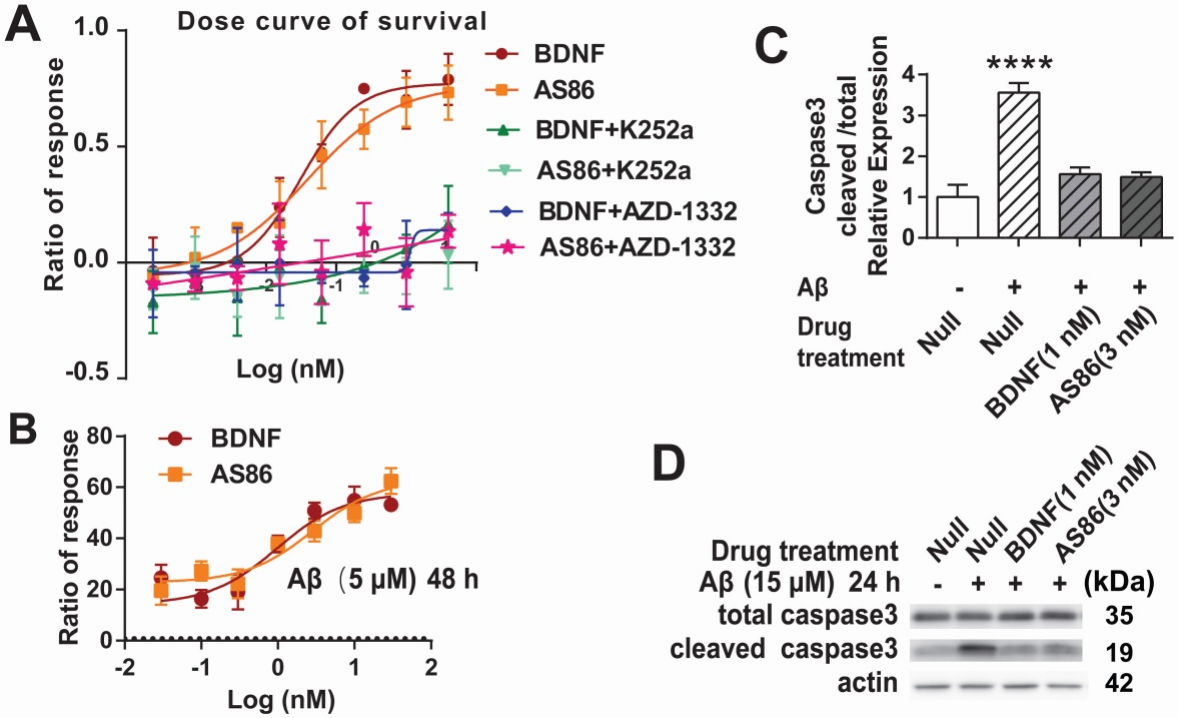
** Cell survival function of AS86.** (**A**) Attenuation of serum-deprivation induced cell death by AS86. Different doses of BDNF or AS86 were applied to serum-deprived cultures of hTrkB-PC12 cells in the absence or presence of Trk inhibitors (300 nM K252a or 50 nM AZD-1332) for 16 hours, and the levels of apoptosis were determined by the ratio of the number of caspase 3 positive cells to total number of cells, using a caspase 3-substrate kit. Survival rates were measured by the decreased apoptotic levels normalized to that of vehicle treatment (n = 3). (**B**) Attenuation of Aβ induced cell death by AS86. Hippocampal neurons were pretreated with AS86 or BDNF for 30 minutes, followed by treatment with Aβ (25-35) (5 µM). Cell viability was analyzed with ATP level quantification assay 48 hours later (N = 2 independent experiments, n = 6 samples). (**C, D**) Inhibition of Aβ induced apoptotic signals by AS86. Hippocampal neurons were pretreated with AS86 or BDNF for 30 minutes, followed by treatment with Aβ (25-35) (15 µM) for 24 hours. Total and cleaved caspase3 levels were measured using Western blotting (N = 3 independent experiments, n = 3 replicates). Representative Western blots (D) and quantitative plots (C) are presented.

To determine whether the TrkB agonistic antibody could counter cell death and Aβ toxicity, we used PC12 cells expressing human TrkB. Apoptotic cell death, measured by fluorescent substrate cleaved by caspase-3, was induced by serum deprivation in hTrkB-PC12 cells. The hTrkB-PC12 cells were treated with BDNF or AS86, and the protective effects were examined after the cells were serum-deprived for 16 hours. Both AS86 and BDNF elicited dose-dependent rescue effects, with EC50 at 0.039 nM and 0.031 nM, respectively (Figures 3A and S4A). These effects were blocked by the TrkB inhibitors (K252a or AZD-1332) (Figure 3A). Next, we used an Aβ induced cell death assay to assess the potential of AS86 in Alzheimer's disease (AD) therapy. Hippocampal neurons (DIV10) were treated with Aβ (25-35), the most toxic fragment of Aβ (1-42) peptide, to induce cell death 69, 70. Based on the dose-response curve (Figure S4B), 5 μM of Aβ (25-35) was used to induce moderate cell death. Under these conditions, pretreatment with AS86 or BDNF effectively prevented Aβ (25-35) induced neuronal death and elicited a dose-dependent increase in survival rate, when normalized to the “Aβ (25-35) treated alone” group (Figure 3B). Cleaved caspase 3, an apoptosis marker, was significantly increased after treatment with Aβ (25-35) for 24 hours (3.6 fold, p < 0.0001). This increase was almost completely attenuated if the neurons were pretreated with AS86 (3 nM) or BDNF (1 nM) (Figure 3C-D). Therefore, similar to BDNF, the TrkB agonistic antibody AS86 attenuates apoptotic cell death induced by serum deprivation or Aβ (25-35) through activation of TrkB.

### Facilitation of dendritic growth by AS86

**Figure 4:**
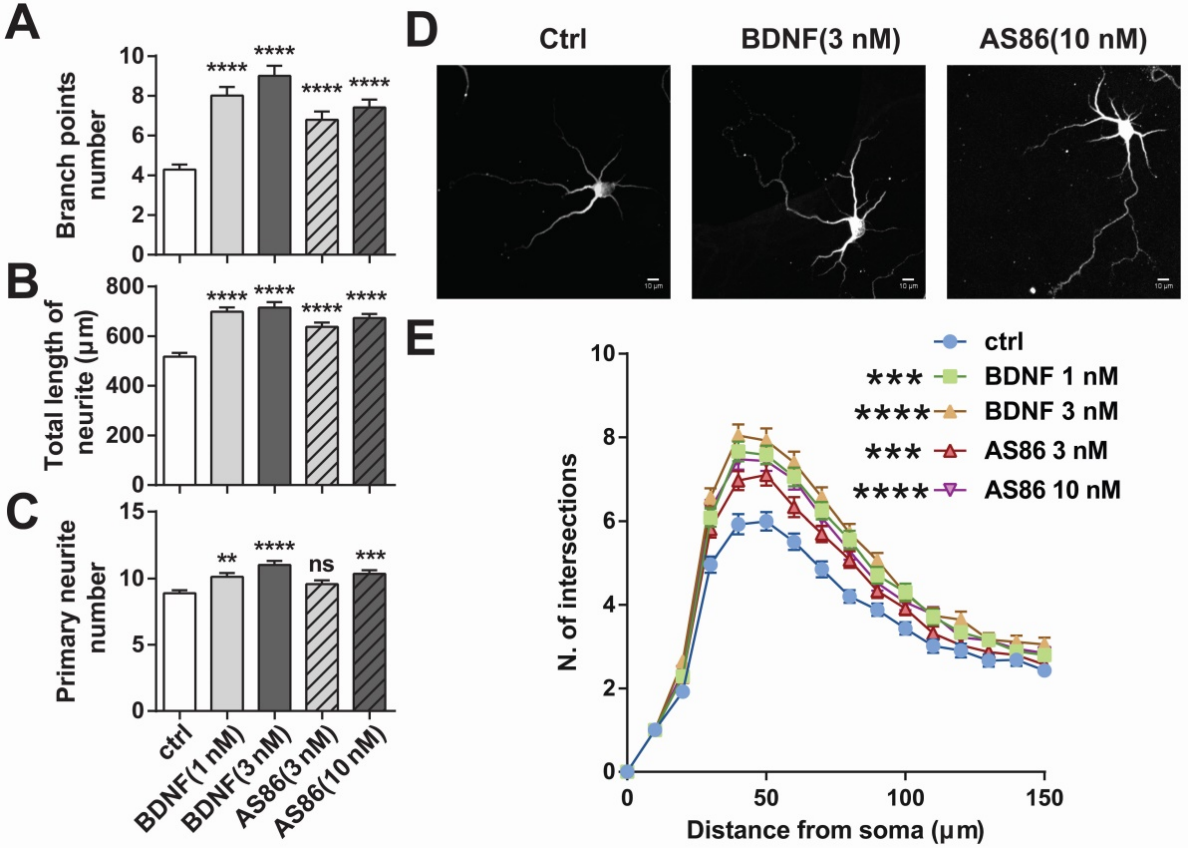
** Differential effects of AS86 and BDNF on neurite growth in hippocampal neurons.** Cultured hippocampal neurons (DIV1) were treated with BDNF or AS86 for 3 days, and then fixed and stained with MAP2 antibody. Quantifications of branching points (**A**), total length of neurites (**B**) and primary neurite numbers (**C**), neuronal images with MAP2 staining (**D**) and sholl analysis (**E**) are presented (N = 3 independent experiments, n = 120-140 neurons). Scale bar represents 10 µm.

BDNF could elicit a variety of cellular functions including promoting neurite growth 27,71. We examined whether AS86 has similar capacities using cultured hippocampal neurons derived from E18 rats. Treatment of hippocampal neurons (DIV1) with AS86 (3 nM, 10 nM) or BDNF (1 nM, 3 nM) for 3 days resulted in a significant increase in both neurite branch points and neurite total length (Figures 4A, B and D). Primary neurite number was also increased by AS86 at 10 nM (Figure 4C-D). Sholl analysis indicated that both AS86 and BDNF increased neurite complexity (Figure 4E).

### Enhancement of synaptic functions by AS86

**Figure 5:**
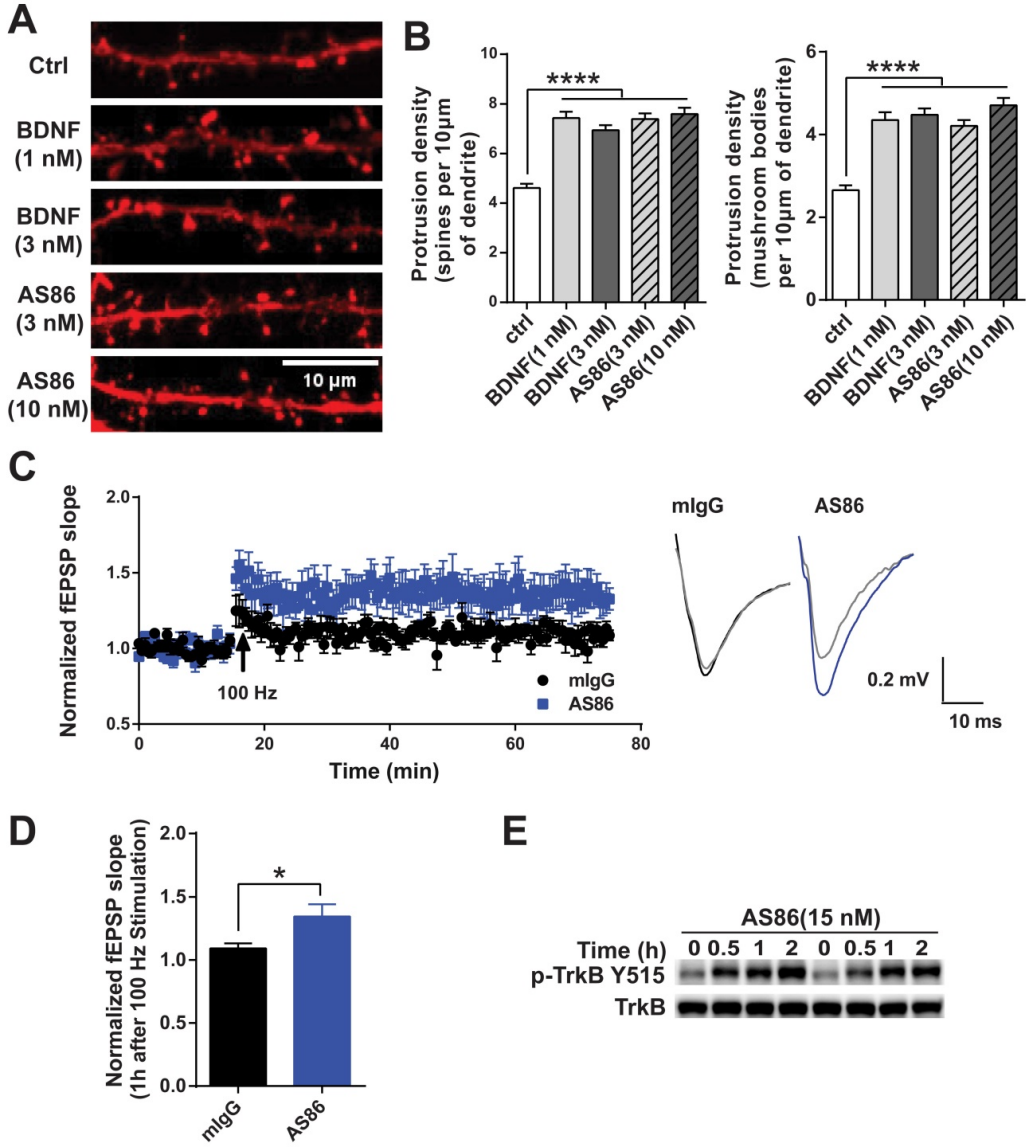
** Facilitation of synaptic functions by AS86.** (**A-B**) Effects of AS86 on dendritic spine growth. Cultured hippocampal neurons (DIV7) were transfected with mCherry, followed by treatment with AS86 or BDNF at DIV 15 for 24 h before fixation (N = 3 independent experiments, n = 60 dendrites). Images of dendritic spine (**A**) and the quantifications of dendritic protrusion densities (**B**) are presented. Ctrl in this and all other figures: Control, no treatment. (**C**) Enhancement of HFS-induced LTP in hippocampal slices treated with AS86. AS86 or mIgG was added into perfusate at least 30 min before LTP induction and maintained in perfusate during entire course of recording (65 min). Field EPSPs (fEPSPs) were recorded and the slopes of fEPSPs were plotted over time. The right panels show the representative fEPSPs before HFS (gray) or after stimulation (blue or black). (**D**) Quantification of slope of fEPSPs 1 hour after LTP induction (n = 7 slices) (Student's *t*-test). (**E**) Activation of TrkB by AS86 in hippocampal slices. P12 hippocampal slices were treated with AS86 (15 nM) for 0.5, 1 and 2 hours, and the lysates were analyzed by Western blotting using anti-pTrkB antibody Y515.

Numerous studies have demonstrated that BDNF promotes dendritic spine growth and facilitates synaptic transmission and plasticity 25,27,28. Thus, BDNF-mediated synaptic repair has been proposed as a new therapeutic strategy for neurodegenerative diseases including AD 10. We examined whether AS86 could mimic BDNF on synaptic plasticity. Hippocampal neurons expressing fluorescent protein mCherry (red) were grown for 15 days in culture and treated with AS86 (3 nM or 10 nM) or BDNF (1 nM or 3 nM) for 24h, and neuronal images were examined by the confocal microscope. Similar to BDNF, AS86 significantly increased dendritic spine density (control = 4.6 ± 0.16, AS86 3 nM = 7.4 ± 0.23, AS86 10 nM = 7.6 ± 0.25). In particular, the density of mature, mushroom-shaped spines was markedly increased (control = 2.7 ± 0.12, AS86 3 nM=4.2 ± 0.14, AS86 10 nM= 4.7 ± 0.17) (Figure 5A-B; all p < 0.0001).

We have previously shown that exogenous BDNF facilitates LTP (long-term potentiation) in slices derived from young postnatal day 12-13 (P12-13) hippocampus in which endogenous BDNF is low 25. Here we performed a series of experiments to determine whether AS86 could mimic the effects of BDNF on LTP using rat hippocampal slices. Time course and dose-response experiments indicated that treatment of hippocampal slices derived from P12 rats with 15 nM AS86 for 2 hours elicited a robust activation of TrkB (Figures 5E and S5A). Among all slices derived from rats of different ages (P10-P28) treated with AS86 under these conditions (2 hours, 15 nM), P12 slices exhibited the most robust activation of TrkB and its downstream signals such as Akt and Erk (Figure S5B). We therefore used hippocampal slices from P12-P13 rat for the LTP experiments.

Field excitatory postsynaptic potentials (fEPSPs) in the CA1 region of hippocampal slices were recorded in response to stimulation of the Schaffer collateral pathway. Similar to our previous observation 25, in P12-13 hippocampal slices of rats, high frequency stimulation (HFS, 100Hz, 1 sec) can only induce a subthreshold, small LTP (109 ± 3.9%, n = 7 slices) in control slices which were pretreated with 15 nM mouse IgG (mIgG). In contrast, in slices pretreated with AS86 (15 nM, 30 min), a HFS-induced stable LTP was observed (134 ± 9.6%, n = 7 slices) (Figure 5C-D). The slope of fEPSPs in AS86 treated slices 1 hour after HFS was significantly enhanced compared with that of mIgG treated slices (Figure 5D; p = 0.0328). However, AS86 administration did not change the input-output curve (Figure S5D). Similarly, in paired-pulse facilitation (PPF) test, the bath application of AS86 (15 nM) did not change PPF at different time points (Figure S5C). These results suggest that AS86 had no effect on basal synaptic transmission. The ability of AS86 to facilitate dendritic spine growth and LTP, together with its role in promoting neurite outgrowth and countering Aβ-induced cell death, forms the basis for further investigation of the therapeutic potential of AS86 for AD.

### Target engagement of AS86 in mouse brains

**Figure 6:**
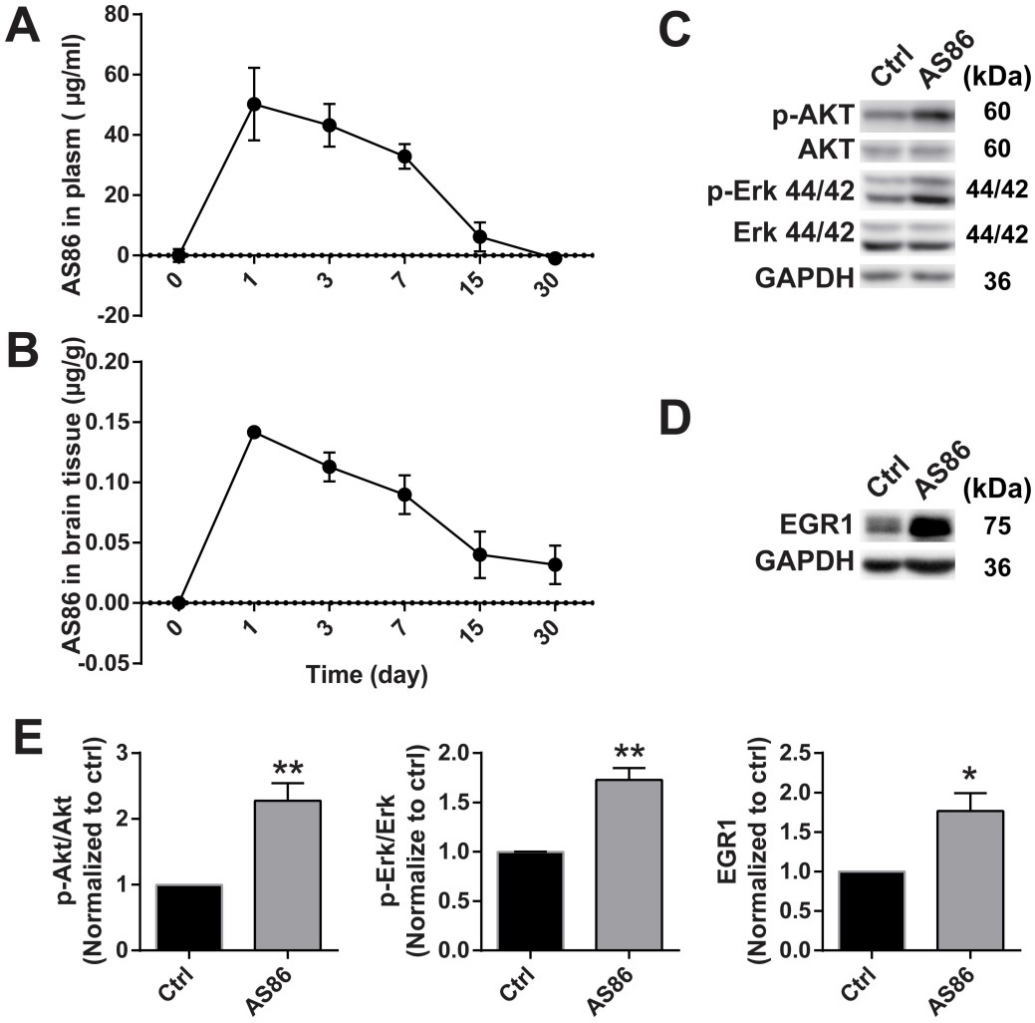
** Target engagement of AS86 in mouse brains.** (**A-B**) The pharmacokinetic curves of AS86 in plasma and brain tissues of mice. Mice were injected with AS86 (1.5 mg/kg) by tail vein injection, and the AS86 concentration at different time points were analyzed with ELISA, and plotted in time-curve graphs for plasma (A) and brain tissue (B) respectively (n = 3-4 mice). (**C-E**) Activation of TrkB downstream signals and gene expression by AS86. The hippocampal tissues were lysed after AS86 (1.5 mg/kg) was administrated through tail vein injection, and p-Akt, p-Erk, and EGR1 expression were analyzed at 1 day after injection (n = 3 mice). Representative Western blots (C, D) and quantitative plots (E) are presented (Student's *t*-test).

To investigate the therapeutic effects of AS86 on AD *in vivo*, it is important to determine whether AS86 could engage its target TrkB in the mouse brain. We determined the pharmacokinetics of AS86 in blood and brain, and whether it can penetrate blood-brain barrier (BBB) when administered peripherally. We administered AS86 (1.5 mg/kg body weight) to mice through tail vein injection and collected the plasma and brain tissues (after perfusion) at different time points. AS86 concentrations were examined and quantified by ELISA. We found that AS86 had a half-life of approximately 6 days in both mouse plasma and brain. AS86 was almost eliminated completely in blood by 30 days but remained at approximately 20% levels in the brain (Figure 6A-B).

Body weight reduction cannot be a direct pharmacodynamic (PD) marker for the main effects we examined in this paper - the amelioration of cognitive impairments, which are largely mediated by cortical and hippocampal synapses. However, substantial evidence supports the view that BDNF-mediated body weight reduction is achieved by enhancement of synaptic function in the hypothalamus. Given the difficulties in finding a direct PD marker for brain diseases, a common strategy in CNS drug development is to find a surrogate marker linked to the key mechanism of the drug. Previous studies showed that treatment with TrkB agonistic antibodies resulted in weight loss 72, which is mainly caused by inhibition of food intake through enhancement of synaptic function by BDNF-TrkB signaling in the hypothalamus. Therefore, body weight could be used as a surrogate indicator for the PD measurement. We found that body weight reduction began from day-1 after a single-dose administration of AS86 and continued to a plateau level by day-7, and began to recover after day-14 (Figure S6A). The body weight always remained at a reduced level after repeated doses of AS86 (Figure S7C), revealing a PD profile for single dose AS86. We also examined the downstream signaling and the expression of EGR1, an early response gene downstream of TrkB activation. AS86 increased p-Akt, p-Erk and EGR1 expression (Figure 6C-E). These results suggest that AS86 can pass the BBB and penetrate into mouse brain.

### Effect of AS86 on novel object recognition

**Figure 7:**
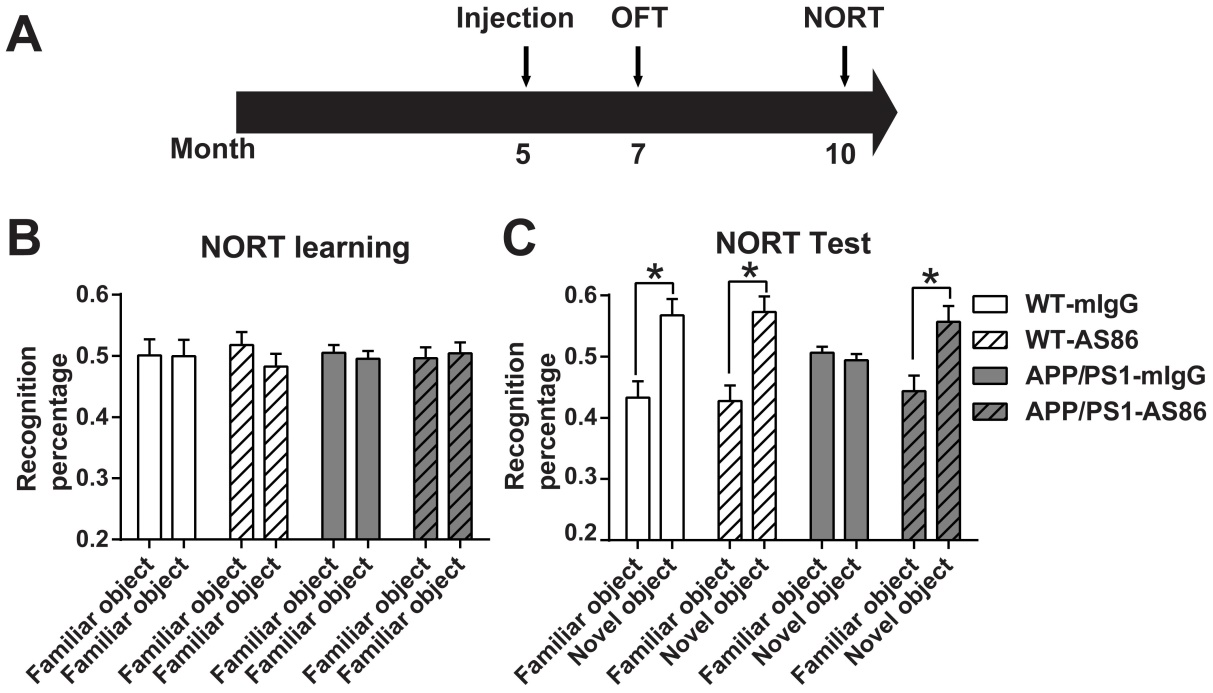
** Effects of AS86 on novel object recognition test (NORT) in APP/PS1 mice**. (**A**) Schematic diagram showing the timeline of drug treatments and behavior tests were performed. (**B-C**) Performance of NORT behavior after AS86 treatment. Mice were subjected to NORT after AS86 (1 mg/kg) treatment for 5 months. WT-mIgG (n = 7), WT-AS86 (n = 10), APP/PS1-mIgG (n = 10) and APP/PS1-AS86 (n = 15) mice were subjected to learning paradigm (B), followed by the test trail (C) 3 hours later. Note that administration of AS86 significantly improved the ability to recognize novel objects by APP/PS1 mice. Paired student's *t*-test.

Previous studies have shown that BDNF injected to the brains of an AD mouse model could improve cognitive functions 44, 48. Here we tested whether the TrkB agonistic antibody AS86, with significant improvements in PK and BBB penetration over BDNF shown above, could induce behavioral changes when administered peripherally to APP/PS1, a commonly used AD mouse model. The littermates APP/PS1 were divided into four groups: Wild-type (WT)-mIgG, WT-AS86, APP/PS1-mIgG and APP/PS1-AS86. AS86 or mIgG was administered into 5-month-old mice through tail vein injection every two weeks (Figure 7A). The peak value of antibody concentration in homogenate of brain tissue is about 1 nM (0.15 μg AS86/g brain weight) according to the PK analysis with the single dose of 1.5 mg/kg. In fact, the antibody cannot penetrate through cell membranes. Therefore, the actual concentration in brain was estimated to be about 3-5 nM, which could elicit more than 80% TrkB activation and promote cell survival *in vitro*. Based on the PK analysis (Figure 6B), there should still be approximately 30% AS86 left at 15-30 days, after a single dose of AS86. Considering the cumulative effect induced by repeated dosing with an interval of 2 weeks, we chose the dosage of 1 mg/kg body weight for our behavioral experiments.

Consistent with previous reports 72, administration of AS86 resulted in a moderate loss of body weight (20%, Figure S7C). To determine whether the weight loss altered spontaneous motor activities, we performed the open-field task using 7-month old animals (AS86 treatment for 2 months). There were no significant differences in velocity, moving distance, time spent in center and time spent in corner among these four groups (Figure S7A; all p > 0.05). It has been demonstrated that BDNF may induce hyperalgesia in intact animals 73. Therefore, we performed experiments of von Frey and Plantar test to examine the cutaneous sensitivity 1-day after AS86 treatment. Treatment with AS86 elicited no effect on sensory functions as determined by these tests. (Figure S6B-C).

We first examined the effect of AS86 treatment on novel object recognition test (NORT) using 10-month old APP/PS1 mice with 5-month treatment of AS86. We found that AS86 treatment had no effect on NORT learning of two familiar objects both in WT and APP/PS1 mice (Figure 7B). Moreover, long-term treatment with AS86 did not further improve NORT memory of control animals (Figure 7C). WT animals spent approximately 20% more time on novel object than familiar object, regardless whether they were treated with AS86 or not (Figure 7C; the first 4 columns). However, in the test phase, the APP/PS1 mice exhibited a severe impairment in NORT scores. APP/PS1 mice treated with mIgG spent equal amount time on familiar and novel objects (Figure 7C; Compare the 5^th^ and 6^th^ columns, p = 0.9124, paired student's *t*-test). In marked contrast, APP/PS1 mice treated with AS86 for 5 months exhibited huge preferences towards novel object, almost the same level as the WT animals (Figure 7C; Compare the 7^th^ and 8^th^ columns, p = 0.0113, paired student's *t*-test). These results indicate that long-term (5 months) treatment of APP/PS1 mice from 5 months to 10 months old is efficacious in rescuing the deficits in novel object recognition.

### Effects of AS86 on spatial learning and memory

**Figure 8:**
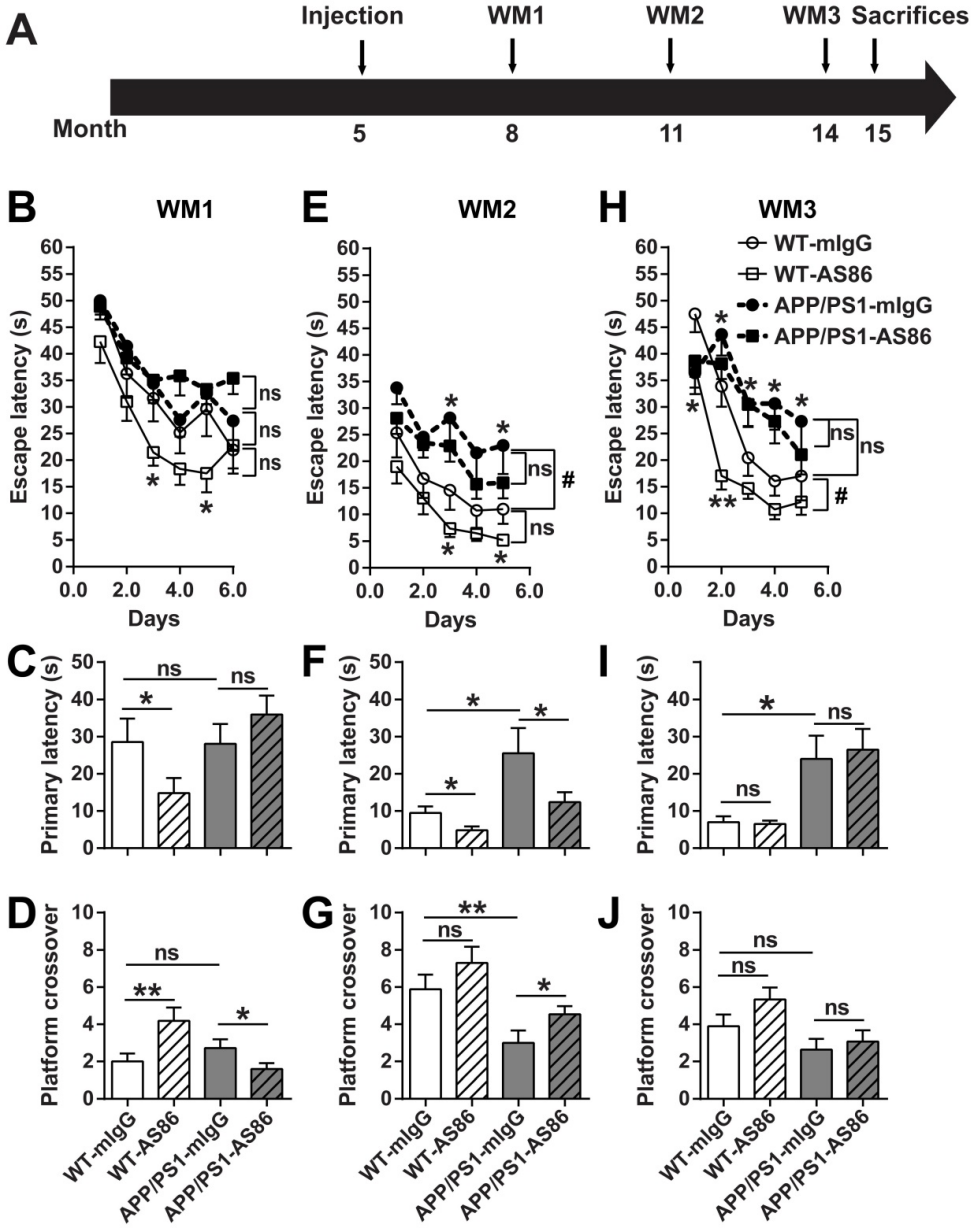
** Effects of long-term treatment of AS86 on spatial learning and memory in APP/PS1 mice.** (**A**) Schematic diagram showing the time point when AS86 was first dosed as well as those when Morris water maze (WM) tests were performed. Mice were subjected to tail-vein injection with AS86 or mIgG every two weeks for 10 months (5 to 15 months old). Three tests of WM were performed during this period. (**B-J**) Morris water maze (WM) tests were performed to evaluate the effects of AS86 (1 mg/kg) treatments for different durations. (B-D) First evaluation: AS86 (1 mg/kg) treatment for 3 months (WM1). WT-mIgG (n = 10), WT-AS86 (n = 11), APP/PS1-mIgG (n = 14) and APP/PS1-AS86 (n = 18) mice were subjected to learning paradigm of water maze for 5 days, followed by a probe trial at day 6. (E-G) Second evaluation: AS86 treatment for 6 months (WM2). WT-mIgG (n = 9), WT-AS86 (n = 11), APP/PS1-mIgG (n = 12) and APP/PS1- AS86 (n = 18) mice were examined in this round (4 days of training trails, and followed by a probe trial at day 5). (H-J) Third evaluation: AS86 treatment for 9 months (WM3). WT-mIgG (n = 9), WT-AS86 (n = 9), APP/PS1-mIgG (n = 10) and APP/PS1- AS86 (n = 15) mice were subjected to WM (4 days of training trails, and followed by a probe trial at day 5). Statistics for the escape latency of learning trails (B, E, H): Two-way ANOVA with repeated measurement was used for the learning curves. Significant differences in WM2 (WT-mIgG and APP/PS1-mIgG) and WM3 (WT-mIgG and WT-AS86) were indicated by bracket plus “#” (p < 0.05). ns: not significant. Student's *t*-test was for individual day comparison. For data points significantly different from the corresponding point in WT-mIgG. *: p < 0.05; **: p < 0.01. Statistics for the primary latency (C, F, I) and platform crossover times (D, G, J) of probe trails: the results of two-way ANOVA (mIgG. vs. AS86, and WT. vs. APP/PS1) were shown in Table 1, 2. For the comparison between specific groups, the independent-samples *t*-test or non-parametric (Mann-Whitney) test was selected depending on whether the data followed normal distribution. *: p < 0.05; **: p < 0.01. ns: not significant.

The NORT results suggested that long-term treatment of AS86 by tail vein injection could improve cognition in APP/PS1 mice. Next, the same mouse model was used to investigate the effect of AS86 on spatial memory deficits, a form of impairment most relevant to AD. Morris water maze (WM), which could be tested repeatedly when the platform location changed, was used. AS86 was administered twice monthly to APP/PS1 mice beginning at 5 months of age, and WM tests were performed 3 times (WM1, WM2 and WM3) when the animals were at the age of 8, 11, and 14 months (with drug treatments for 3, 6, and 9 months), respectively (Figure 8A).

In WM1, the mice were trained for 6 days. The escape latency for the WT-mIgG group in last three training trials on the 6th day reached the test standard (20s). We found that there was no difference in spatial learning and memory between the WT and AD mouse model (WT vs. APP/PS1 groups, all p* >* 0.05, Figure 8B-D, Table 1, 2), indicating that APP/PS1 had not developed spatial cognition deficiency at the age of 8 months. Treatment of the WT animals with AS86 for 3 months slightly enhanced the learning performance (Figure 8B, compare WT-mIgG with WT-AS86 groups; F (1, 19) = 4.356, p = 0.0506), and this enhancement reached statistical significance on day 3 (p *=* 0.0310) and day 5 (p = 0.0366). In the probe test on day 7, treatment with AS86 elicited no effect in primary latency and platform crossover (Table 1, 2), compared with mIgG group. However, WT-AS86 group also exhibited better scores than WT-mIgG group in the primary latency (Figure 8C; p = 0.0436) and the platform crossover number (Figure 8D; p = 0.0093). These results suggest that AS86 could enhance cognitive functions of WT mice. Treatment of APP/PS1 with AS86 for the same duration (3 months) generally did not improve spatial learning and memory (Figure 8B-D). Paradoxically, the APP/PS1-AS86 group performed worse compared with the APP/PS1-mIgG group in the platform crossover (Figure 8D; p = 0.0262).

In WM2, AS86 appeared to be most effective in improving cognitive functions for both WT and APP/PS1 mice. First, APP/PS1-mIgG group showed a dramatic deficit in spatial learning compared with WT-mIgG (Figure 8E; compare WT-mIgG with APP/PS1-mIgG groups; F (1, 19) = 4.905, p = 0.0392; day 3, p *=* 0.0166, day 5, p = 0.0450). The probe test also revealed a marked impairment of APP/PS1-mIgG group, compared with WT-mIgG group, in spatial memory, as reflected by the increases in primary latency (Figure 8F; p *=* 0.0270) and platform crossover number (Figure 8G; p *=* 0.0063). Second, treatment with AS86 for 6 months improved the spatial learning for WT (Figure 8E, compare WT-mIgG and WT-AS86 groups; F (1, 18) = 3.936, p = 0.0627; day 3: p *=* 0.0360, day 5: p = 0.0180) and exhibited a non-significant trend of improvement in ASP/PS1 mice (Figure 8E, compare APP/PS1-mIgG and APP/PS1-AS86 groups: F (1, 28) = 1.514, p = 0.2287). Most importantly, AS86 rescued the spatial memory deficiency of APP/PS1 mice in the primary latency (Figure 8F; p *=* 0.0464) and platform crossover (p = 0.0274). For WT mice, AS86 treatment improved the primary latency (Figure 8F, p = 0.0164) but not platform crossover (Figure 8G, p = 0.1268). Similar conclusions could be reached using two-way ANOVA analysis of the probe test data (Table 1, 2).

In WM3, 3 months after WM2, regardless whether the animals were treated with AS86 or not, the APP/PS1 exhibited poorer performances in spatial learning and memory than WT mice (Figure 8H-J, Table 1, 2). In animals of this age (14 months old), long-term treatment with AS86 (9 months) did not show any effect on spatial memory compared with mIgG group (Table 1, 2). AS86 failed to rescue the impairments in spatial learning (Figure 8H) or memory (Figure 8I-J) in APP/PS1 mice (all p *>* 0.05). For comparison, treatment of WT mice with AS86 also showed an improvement in spatial learning (Figure 8H, compare WT-mIgG with WT-AS86 groups; F (1, 16) = 8.282, p = 0.0109; day 1 p = 0.0373, day 2 p = 0.0011) and a small but non-significant effect on platform crossover (Figure 8J, p = 0.0649). However, treatment with AS86 seemed to enhance the lifespan of both WT and APP/PS1 mice (Figure S7B).

Given that AS86 treatment resulted in a moderate decrease in body weight, we examined whether body weight differences affected the performance of WM test. We analyzed the data from all groups and found there is no correlation between body weight and primary latency or platform crossover number (Figure S7D; all r square < 0.95, all p > 0.05, Linear Regression). Moreover, no difference in swimming velocity was found compared with WT-mIgG group (Figure S7E; all p > 0.05).

The WM experiments together suggested that activation of TrkB by AS86 administration seemed to improve spatial learning and memory slightly for WT but more so for the APP/PS1 mutant mice. In the AD model, treatment with AS86 elicited no effect before spatial memory deficiency (8-month old mice, treated for 3 months), significant effects at the early stage of spatial memory deficiency (11-month old mice, treated for 6 months), and no effects at the middle stage of memory deficiency (14-month old mice, treated for 9 months). Thus, the age of animals (stage of disease manifestation) and duration of AS86 are both important. Treatment of younger animals (8-month old, presumably at the early stage of AD) with AS86 for 3 months seems to be marginally effective. In older animals (14 months or older) in which the Aβ pathology becomes overwhelming, activation of TrkB would no longer be effective regardless of how long the TrkB agonist is administered.

### Effects of AS86 on pathology and pathophysiology of AD mice

**Figure 9:**
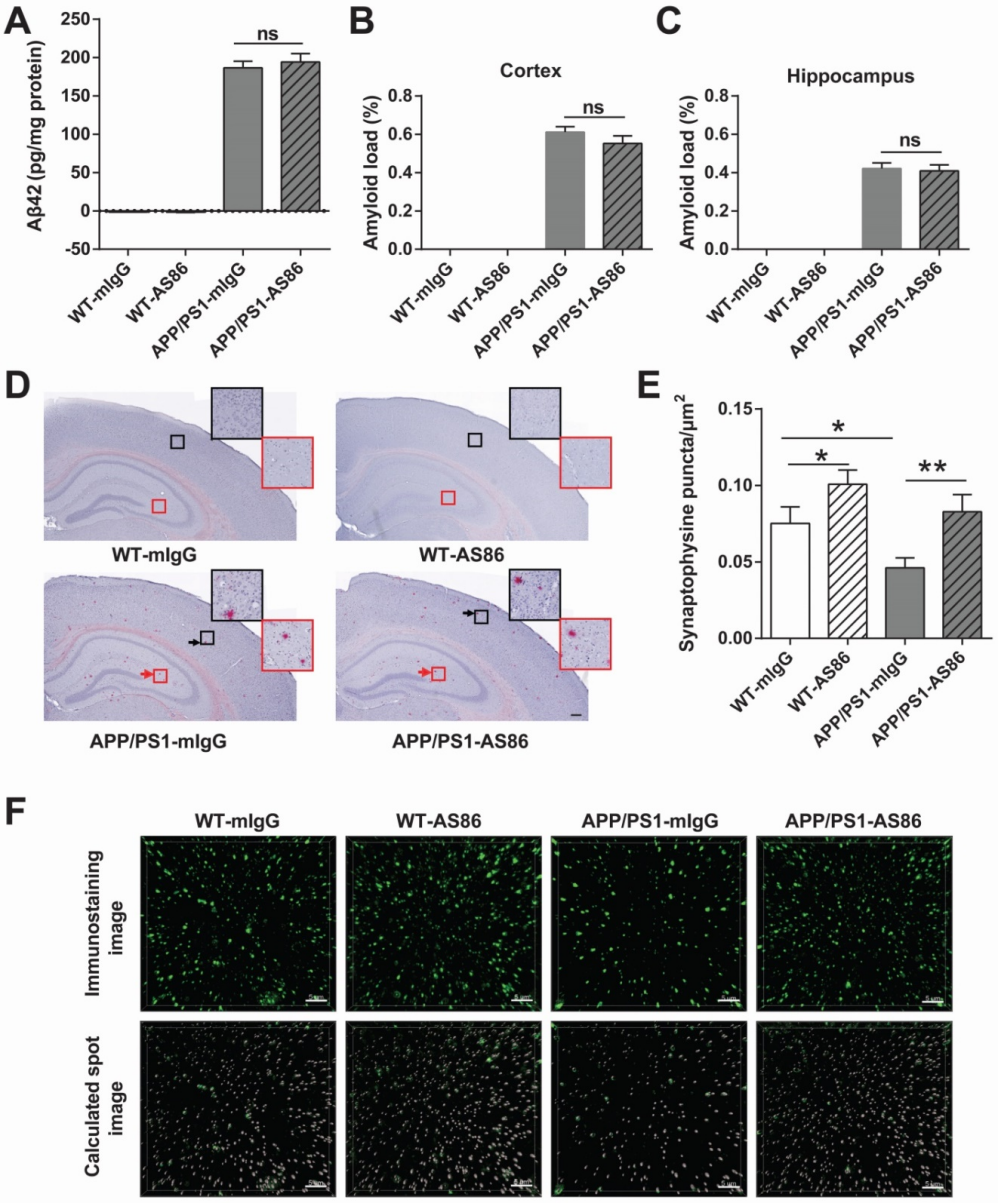
** Effects of AS86 on pathology and pathophysiology of AD mice.** (**A-D**) Effect of AS86 treatment on Aβ pathology in APP/PS1 mice. (A) Detection of Aβ42 for each sub-group. Human Aβ42 in forebrain (15 months) was measured with Aβ42 Elisa (n = 6 for each group), followed by quantification. (B, C, D) Amyloid plaque load in hippocampus and cortex. Amyloid plaques were detected by Congo red staining in hippocampus and cortex (12 slices from 6 mice for each group). The quantification of amyloid plaque load percentage in cortex (B) and hippocampus (C) and the Congo red staining images (D) are presented. Scale bar represents 200 µm. The black and red arrows indicated the amyloid plaques in cortex and hippocampus respectively. The images in black and red squares were amplified at the upper right. (**E, F**) Rescue of synaptophysin loss in APP/PS1 mice by AS86. Synaptophysin puncta in hippocampal CA1 (15 slices from 6 mice of 15 months old for each group) were detected with immunohistochemistry. The density of synaptophysin puncta in each group was analyzed blindly by Imaris software with the same standard. The immunostaining images (upper) and calculated spot images (lower) with Imaris software (F), and the histogram of quantification for puncta numbers are presented (E). Scale bar represents 5 µm. Student's *t*-test.

A hallmark of AD is the aggregation of Aβ42 peptide, leading to accumulation of amyloid plaques in the brain 7,74. In APP/PS1 mice, the Aβ pathology occurs at around 6 months of age, and in 12-month old animals, amyloid plaques are seen throughout the brain 75,76. We examined whether long-term treatment with AS86 could alter Aβ pathology in APP/PS1 mice. AS86 was administered twice monthly through tail vein injection to the APP/PS1 mice at 5 months of age, and Aβ42 level in forebrain was examined with ELISA after 10 months of treatment with AS86. As shown in Figure 9A, long-term treatment with AS86 had no effect on the Aβ42 level in APP/PS1 mice (p > 0.05). Next, we examined the amyloid plaques in the brain using Congo red staining method. The percentage of amyloid load in hippocampus and cortex were quantified. Again, a 10-month treatment with AS86 did not change amyloid load either in hippocampus or in cortex (Figure 9B-D; all p > 0.05). These results suggest that improvement of cognitive functions by AS86 was not mediated by a reduction of Aβ pathology.

A key pathophysiological feature of AD brain is synaptic loss 11-13. To determine whether AS86 attenuated cognitive impairment in APP/PS1 mice by synaptic repair, we examined the presynaptic marker protein synaptophysin in CA1 area of hippocampus. After 10-month treatment with AS86 by tail vein injection, the density of synaptophysin puncta was analyzed. Among the four groups of mice, the synaptophysin puncta density of APP/PS1-mIgG mice was significantly decreased compared with WT-mIgG mice (p = 0.0139). In contrast, the APP/PS1-AS86 group exhibited a significant increase in synaptophysin puncta density compared with APP/PS1-mIgG group (p *=* 0.0054) (Figure 9E-F). There was also a significant increase in WT-AS86 group (p *=* 0.0398) compared with WT-mIgG group. Therefore, AS86 treatment ameliorated synaptic loss as reflected by the synaptophysin puncta density in the 15-month-old APP/PS1 mice.

## Discussion

Despite plenty of evidence for the role of Aβ in the pathogenesis of Alzheimer's disease, therapies aimed at reducing Aβ accumulation have so far been unsuccessful. A paradigm shift is needed to treat the devastating illness. A large body of literature now supports the view that synapse loss, but not toxin accumulation, correlates with disease progression 2,7. Thus, an emerging new approach is to address AD using neuronal and/or synaptic repair mechanisms. BDNF, the best known “synaptogenic” molecule demonstrated by animal studies and human genetics, may pave the way for this paradigm shift. However, decades of R&D efforts by many biopharmaceutical companies have suggested that BDNF protein itself may not be a good drug candidate due to its intrinsic properties. Here we have identified a TrkB agonistic antibody AS86 that could effectively penetrate into the brain and activate the BDNF-TrkB signaling pathways in the central nervous system (CNS). We provide a set of coherent evidence that AS86 could inhibit Aβ-induced neuronal death, promote spine formation, and facilitate LTP at hippocampal CA1 synapses. The antibody has also been shown to have a long blood half-life, good brain penetration, and sufficient target engagement. Most importantly, long-term treatment with AS86 attenuated the deficits of recognition and spatial memories at the early stage of spatial memory deficiency in a mouse model of AD. Taken together, the present study has laid a foundation for a new therapy for AD, based on the use of TrkB agonistic antibody.

The idea of targeting the BDNF-TrkB is not new. Several groups have attempted to infuse the BDNF-expressing viruses or BDNF itself into the brains of AD animal models 44,46,48,77,78. While useful in proof of concept (PoC), the methods of delivering viruses or protein directly into the brain would be difficult to implement in the clinic, and acceptance by patients and doctors would be low. Daily administration of a BDNF-mimicking peptide has also been shown to improve memory deficits in rodent models of AD 49. However, the pharmacokinetics of the peptide was not known and cost of goods (CoG) for daily injection of the peptide could be extremely high. Several papers have claimed that small molecule TrkB activators could mimic BDNF in cellular assays and therefore be potentially useful in treating neurodegenerative diseases including AD 62-66. However, two recent studies using more systematic approaches and rigorous methods have reported contrary results: these small molecules neither bind nor activate TrkB 67,68. Based on the structural model of TrkB 79, these compounds are too small to hold the two TrkB monomers together for TrkB dimerization, which is required for the activation of receptor tyrosine kinase of TrkB. To this end, bivalent macromolecules such as monoclonal antibody would be more appropriate for dimerization and activation of TrkB. Several TrkB agonistic antibodies have been developed in the past 67,80,81. However, treatment of chronic illnesses such as AD would require long-term drug administration that demands a high bar for safety, and none of the previously reported TrkB antibodies have been tested in AD animal models.

The present study is the first to systemically examine whether long-term administration of TrkB agonistic antibody could be a feasible approach for AD therapy. We show that AS86, a monoclonal TrkB agonistic antibody, exhibited several features superior to BDNF: it binds TrkB but not p75^NTR^ (Figure 2A), is more likely to be diffusible in brain tissues 82, and has a much longer half-life (about 6 days) in blood and brain (Figure 6A-B). In a TrkB activation assay with saturated concentration of BDNF, AS86 exhibited non-competition with BDNF suggesting that AS86 may elicit additional benefits in the presence of endogenous BDNF *in vivo* (Figure S2A-B). In addition to its non-competitiveness with BDNF, we selected AS86 among many other TrkB-agonistic antibodies as a drug candidate for AD for a number of reasons. First, although TrkB activation induced by AS86 is lower than by BDNF (Figure 1B), the two TrkB activators elicited similar levels of downstream signaling, especially ERK and Akt (Figure 1B-C, Figures S2A, S2C), the two signaling pathways thought to be more important for cell survival and synaptic growth 24. Second, AS86 performed better than other TrkB agonistic antibodies that induced higher TrkB activation (data not shown), but similarly to BDNF in regulating neuronal survival (Figure 3), neurite growth (Figure 4) and synaptic functions (Figure 5). Third, TrkB over-activation may induce potential side-effects during long-term treatment for AD.

Behaviorally, bi-weekly administration of AS86 through tail vein injection for more than 5 months attenuated deficits in spatial as well as recognition memory in a mouse model of AD (Figure 8F-G; Figure 7C), without any impact on motor behavior (Figure S7A). It appears that the drug-induced improvements in spatial memory were not as robust as those on object recognition memory, raising the possibility that AS86 may preferentially alter synapses in various cortical areas over hippocampus. However, it should be noted that hippocampus is also a key area involved in recognition memory. Although we only examined the changes in synaptophysin puncta in the hippocampus, it is our belief that AS86 ameliorated synaptic deficits in hippocampus as well as various cortical regions. In this study, we focused on AS86-induced changes in the hippocampus because we performed spines and LTP analyses in this area. Many previous studies using APP/PS1 mice have also used hippocampus to examine synaptic changes 83. Future studies should be designed to address why the TrkB agonistic antibody affects water maze test and object recognition test differentially, and whether AS86 could also rescue synaptic impairment and improve synapses in the cortex. Interestingly, long-term treatment with AS86 even elicited a slight increase in lifespan (Figure S7B). We further observed that bi-weekly administration of AS86 for as long as 9 months did not induce any visible side-effects. Taken together, we have developed a TrkB-specific agonistic antibody as a drug candidate potentially useful for the treatment of Alzheimer's disease in humans.

It is well established that BDNF in hypothalamus regulates energy metabolism including feeding. Several studies have also shown that treatment with TrkB agonistic antibody resulted in a gradual weight loss in mice 72. Therefore, TrkB agonistic antibody had been considered as a potential drug for obesity. We found that long-term treatment with AS86 resulted in a 20% reduction of body weight in the first 20 days, but the body weight maintained unchanged in the next 9 months (Figure S7C). Interestingly, both WT and AD mice exhibited the same magnitude and time course of weight reduction. In general, this reduction in body weight is considered not harmful to health. More importantly, our own data indicate that this body weight loss did not influence the velocity, moving distance, or the time spent in center and corner in the open field task (Figure S7A). On the contrary, mice may benefit from the weight loss according to the preliminary data on the survival curve shown in Figure S7B. Thus, the mild reduction in body weight may be tolerable even for aged animals.

A major challenge for the treatment of CNS diseases with antibody drugs is whether they can effectively penetrate into the brain and engage their targets, although several techniques have been established to enhance brain delivery of antibody drugs 84,85. It is believed that the BBB is slightly leaky in the AD brain. Based on data from rat studies, it is estimated that approximately 0.1% - 0.2% of the antibody could pass through the BBB 86. Clinical studies using anti-Aβ antibodies also demonstrate the penetration through BBB and functions of antibodies in the brain through peripheral treatment. In the present study, we show that administration of AS86 at 1.5 mg/kg (body weight) through tail vein injection could effectively activate TrkB and its downstream signaling in the brain (Figure 6C-E). The pharmacokinetic curve of AS86 in brain is matched to that in plasma, and the half-life of AS86 in brain is a little longer (Figure 6A-B).

An interesting observation from the water maze experiments was that bi-weekly administration of AS86 to APP/PS1 mice beginning at 5-months old was ineffective at 3-month treatment (WM1), but effective at 6-month treatment (WM2), and ineffective again at 9-month treatment (WM3). While the mechanisms underlying this biphasic curve remain to be investigated, a number of points could be discussed. First, in WM1, the lack of efficacy in APP/PS1 mice was not due to the inability of AS86 to reach and engage its target TrkB, because the weight reduction occurred shortly after AS86 administration was initiated (Figure S7C), and AS86 treatment improved learning and memory for WT animals (Figure 8B-D). One may speculate that while AS86 was effective in repairing synapses in the APP/PS1 brains, re-building of the lost connectivity may take longer time to accomplish. Second, in WM2, AS86 treatment enhanced learning for both WT and APP/PS1 mice, and even improved memory for both WT and the mutant mice (Figure 8E-G). One may again speculate that learning and memory may have different sensitivity to AS86. Regardless, a critical window is identified in which the therapeutic effects of AS86 could be clearly demonstrated. These results further suggest the length of clinical trial should not be too short for BDNF-based therapy. Finally, in WM3, continuous treatment with AS86 for 9 months was no longer beneficial for learning or memory in either WT or APP/PS1 mice (Figure 8H-J). However, such treatment was effective in increasing synaptophysin-positive puncta and repairing synapses (Figure 9E-F). Thus, the TrkB agonistic antibody, like many other therapeutic approaches, may not work when the disease is progressed to a very severe stage.

Does activation of BDNF-TrkB signaling inhibit Aβ production? In normal cells or animals, there are several conflicting results on the role of BDNF in APP or Aβ expression 87-90. In one study, Wu et al reported that treatment of APPswe mice with a BDNF-mimetic peptide reduced the levels of Aβ42, Aβ40, the Aβ42/Aβ40 ratio, as well as the protein levels of APP, APP fragments, BACE1 and PS1 91. However, the specificity and the PK of this peptide were not examined, and it is unclear these effects were truly mediated by the BDNF-TrkB signaling pathway. In another study 78, BDNF infusion into lateral ventricles for 6 weeks (0.5 μg, twice a week) decreased Thioflavin-positive Aβ plaques in APP/PS1 mice, although the number of total plaques was unaffected. Given that the reduction in Aβ plaques level by the BDNF was mild (less than 20%), it is less likely that such a reduction would help improve cognition when compared with those by Aβ antibodies 92-94. In marked contrast, Nagahara et al. reported that the expression of BDNF by virus in the brain of J20 transgenic mice did not affect the amyloid plaque density in the hippocampus 44. In addition, some drugs that increase BDNF indirectly also elicit cognitive improvement without reducing Aβ pathology 95,96. Further, transcriptional analyses have revealed that treatment with BDNF could alter the expression of hundreds of genes, many of which are transcription factors 97. Thus, the beneficial effects of BDNF to AD may be achieved through many downstream genes, irrespective of whether it alters Aβ metabolism or not.

In the present study, we found that treatment with AS86 for a prolonged period did not elicit any change in Aβ42 levels or amyloid load, but significantly repair the synaptic deficits and slow down the progression of cognitive impairments in the APP/PS1 model of AD. This is an important finding, because it suggests that disease-modifying therapy of AD could be achieved even in the presence of (or without reducing) Aβ pathology. Further, it opens up the potential of greater benefits of combinational therapies. Future efforts should be directed towards whether a combination of AS86 and the anti-Aβ antibody (such as aducanumab) could achieve a greater therapeutic effect.

A number of important issues remain to be addressed in future research. First, an important feature of Alzheimer's disease is progressive neuronal loss, leading to brain atrophy 14-17. We show that AS86 could rescue Aβ-induced neuronal death in an *in vitro* cell model (Figure 3B-D). Given the lack of obvious neuronal loss in the APP/PS1 model, we could not address whether AS86 is capable of inhibiting neuronal death *in vivo* 98. As far as we know, most if not all Aβ-perturbing animal models published so far exhibit no obvious neuronal death. The neuroprotective function of AS86 should be rigorously tested when such animal models become available. Second, although BDNF could not alleviate the Aβ pathology in APP/PS1 mouse model (Figure 9A-D), an APP-overexpression model, it would be interesting to test whether AS86 will have an effect on Aβ pathology in an APP knock-in model. Finally, synaptic loss and neuronal degeneration are general features that occur in many neurodegenerative diseases, including Huntington's disease 99 and Parkinson's disease 100. It will be interesting to test whether AS86 could be useful in treating other neurodegenerative diseases.

## Conclusion

We have developed a TrkB agonist antibody AS86 which specifically activates TrkB and its downstream signaling, enhances neurite outgrowth and cell survival in *in vitro* model. Based on its capacity of synaptic enhancement, AS86 could rescue the deficiency of spatial cognition and novel object recognition in APP/PS1 mouse model. Taken together, these results suggest that AS86 could be further developed as a potential therapeutic agent for AD.

## Materials and Methods

### Antibody and reagents

The antibodies used in this study were against: phospho-Akt (S473, Cell Signaling Technology, 9271), phospho-TrkA (Y490, Cell Signaling Technology, 9141), phospho-TrkA (Y706/707, Cell Signaling Technology, 4621), phospho-TrkA (Y816, Cell Signaling Technology, 4168), Erk (Cell Signaling Technology, 4695), phospho-Erk (T202/Y204, Cell Signaling Technology, 4370), phospho- PLCγ(Y738, Cell Signaling Technology, 2821), PLCγ(Cell Signaling Technology, 2822), TrkB (80E3, Cell Signaling Technology, 4603), Akt (Santa Cruz Biotechnology, H-136), MAP2 (EMD Millipore Corporation, MAB3418), GAPDH (Bioeasy (Beijing) Technology, BE0023), EGR1 (Cell Signaling Technology, 4153), Synaptophysin1 (Synaptic Systems, 101 002), β-actin (CWBIO, CW0096), and Caspase-3 (Cell Signaling Technology, 8610). The reagents included BDNF protein (Sino Biological Inc, 50240-MNAS), Mouse IgG (YEASEN, 3611ES10), and β-Amyloid protein (25-35) (Synpeptide), K252a (Bio Vision, 2013-500), AZD-1332 (Alomeone labs, A-495).

### Production of TrkB agonistic antibodies by the hybridoma technology

The antigen (recombinant TrkB-ECD-hFc) in DPBS was used to immunize BALB/C mice of about 6~8 weeks old via subcutaneous route injections following a procedure of fast immunization. All experiments involving animals in this study were approved by Tsinghua University Committees on Animal Care. After immunization, animals were selected by tittering using ELISA. Immunized mice with the highest titer were sacrificed and lymph nodes were harvested. The lymphoid cells were suspended in DMEM before fusion with a myeloma cell line Sp2/0-Ag14 by PEG (P7306, sigma). The fused cells were cultured in HAT selecting medium (21060-017, Gibco). On day 7 or 10, one-half medium was changed. After 14 days of culturing, hybridoma supernatants were screened for TrkB-specific monoclonal antibodies. ELISA was used to analyze the affinity of the antibodies with both human TrkB-ECD-His and rat TrkB-ECD-His, and NFAT assay was used to select active TrkB agonists. After selection of positive cell pools, sub-cloning was done by limiting dilution. Hybridoma sequencing was performed with the 5'RACE kit (cat.634858, &634859, Clontech).

### Cell culture and treatment

CHO-TrkB cells were grown in standard DMEM (Gibco, 11960-051) containing 10% FBS (Invitrogen, 26400-044) and GlutaMax supplement (Gibco, 35050061). Embryonic hippocampal neurons (embryonic day 18) were dissociated and plated on 100 ng/ml poly-D-lysine-coated 12-well plates at 200,000 cells per well as described previously 101. Neurons were cultured in Neurobasal Medium supplemented with B27 and GlutaMax (Gibco) for 10 d and then processed for biochemical experiments. Dendritic morphology was studied with cultures grown for only 1 d on coverslips (18 mm) at 10,000 cells per coverslip. Spine density and morphology were studied with cultures grown at 100,000 cells per coverslip (18 mm). Cell survival test was performed with cultures grown at 20000 cells per well on black 96well plates (Corning, 3603). All cultures were maintained at 37 °C in 5% CO2 and 95% air.

### NFAT

The procedure was described previously 82.

### Analysis of neurite complexity

Cultured hippocampal neurons at day 1 *in vitro* (DIV1) were treated with TrkB agonist antibody or BDNF and grown for an additional 3 days. The neurons were fixed and stained with a mouse antibody against MAP2 (1:1000, Millipore). Images were acquired with a Zeiss confocal microscope (20 ×, 488-nm laser, LSM710) in a double-blind manner. Four parameters of neurite growth were analyzed using the Image J analysis software (Neuron J plugins and ShollAnalysis plugins). Typically, images of 100 neurons per group were captured, and three independent experiments were performed.

### Western blot analysis

Cells were lysed in the lysis buffer (20 mM HEPES pH 7.4, 150 mM NaCl, 1% CA-630, 1% sodium deoxycholate, 0.1% SDS, 2 mM EDTA and a phospho-protease inhibitor cocktail purchased from Roche) on ice for 30 min. After 10 min centrifugation at 10000 rpm, the supernatants of lysate were subjected to the loading buffer, incubated at 95 °C for 10 minutes and resolved by SDS-PAGE. Proteins were transferred to PVDF membrane for 1.5 hours at 100 V. The membrane was blocked with 5% bovine serum albumin (BSA) for 1 hour at room temperature and incubated with the primary antibodies in blocking solutions at 4 °C overnight before detection with HRP-conjugated secondary antibodies. Chemiluminescence was detected with ECL solution.

### Survival Assay

The assay of serum deprivation model using hTrkB-PC12 cells was described by previous work 82. For AD cell death model, 2 mM β-Amyloid protein (25-35) stock solution was prepared in sterile distilled water, stored at -80 °C, and incubated for 3 days at 37 °C to aggregate oligomers before use. Hippocampal neurons were cultured for 8 d (DIV8). After the neurons were pretreated with TrkB agonist antibodies or BDNF or control medium for 30 minutes, Aβ25-35 oligomer was added for an additional 48 h. Cell viability was determined by the CellTiter-Glo Luminescent Cell Viability Assay (Promega, G7570).

### Analysis of spine density

Hippocampal neurons (7 days *in vitro* (DIV7)) were transfected with membrane-target mCherry by the calcium phosphate method (Takara, 631312) and examined at 16 d *in vitro* using a Nikon confocal microscope (60 × Oil objective at a 1.5 × zoom, 561 nm laser). Each image consisted of a z stack of pictures taken at a depth interval of 0.15 μm and then projected into one image (max intensity). Morphometric quantifications were performed by investigators blind to the experimental condition. Morphometric measurements were performed with Image J. The density of spine and mushroom bodies were calculated by dividing the spine number by the measured length (in 10 μm) of imaged secondary dendritic stretches (longer than 20 μm). In each experiment, 60 secondary dendrites from 30 neurons were analyzed. Three independent experiments were performed.

### Aβ42 quantification

The brain tissues were homogenized in the lysis buffer described as Western blots analysis on ice, and the homogenates were treated with ultrasonic to be lysed thoroughly. Then the tissue lysates were centrifuged at 12000 rpm for 30 min at 4 °C and the supernatants were collected. Before the analysis with ELISA, the sample protein concentrations were determined by BCA protein assay kit (Pierce, 23225) according to the manufacturer's instruction, and normalized by dilution as the lowest one. Aβ42 ELISA kit (Invitrogen, KHB3441) was used to determine the Aβ42 concentration according to the manufacturer's instruction. Optical signals at 450 nm were read on a microplate spectrophotometer (BioTek, CYTATION 5).

### ELISA Assay

Coating protein (TrkB-ECD , p75, TrkA or TrkC) was added into microplate in Coating Buffer (0.1 M Carbonate Buffer, pH 9.6, NaHCO3 8.4 g/L) overnight at 4 °C. The plate was washed with washing buffer (0.05% tween20 in PBS) for 3 times and then blocked with 0.5% BSA in PBS for 1h at RT, followed by 3-time washing. Standard proteins or samples (serum or protein lysates) diluted to suitable concentrations were added into the microplates and incubated for 2 hours at RT. After samples were washed with washing buffer for 3 times, the HRP-conjugated second antibody in 1% BSA was added into the wells and incubated for 0.5 hours at RT. After 3 times of washing, 100 μl chromogen (TMB) was added into each well and incubated for 15 min in dark, and then 50 μl 2 M HCl was added to stop the reaction. Optical signals at 450 nm were read on a microplate spectrophotometer (BioTek, CYTATION 5).

### Animals and housing conditions

The APPswe/PS1dE9 (APP/PS1) mice were generated by Jackson Laboratories (Bar Harbor, ME, USA) 102. Heterozygous male mice of APP/PS1 and their littermate wild type (WT) mice at 4 weeks old with a B6C3F1 background for behavioral tasks were obtained from the Model Animal Research Center of Nanjing University. These mice were randomly assigned into four groups: WT-mIgG (mouse normal IgG), WT-AS86, APP/PS1-mIgG and APP/PS1-AS86. All the mice in other experiments with a C57BL/6J background and Sprague-Dawley (SD) rats were purchased from Beijing Vital River Laboratory Animal Technology Co., Ltd. The animals were maintained in a temperature-controlled room (21 ± 2 °C) with a 12 h light-dark cycle. The ventilation system of mice cages was independent, and food and water were freely available. All these operations were only conducted at the dark cycle. All procedures followed the Institutional Animal Care and Use Committee (IACUC) guidelines provided by Tsinghua University.

### Slice Preparation

P12-13 male SD rats were anesthetized with isoflurane and sacrificed by cervical removal. The brains were rapidly dissected and placed in ice-cold artificial cerebrospinal fluid (ACSF, saturated with 95% O_2_ and 5% CO_2_), containing (in mM): NaCl (125.8), NaHCO_3_ (26), NaH_2_PO_4_ (1.2), KCl (3.1), CaCl_2_ (2.5), MgCl_2_ (1.5) and D-glucose (10). Subsequently, coronal hippocampal slices (400 μm) were prepared in oxygenated ACSF using a Leica VT1000S vibratome (Leica Instruments) at 4-6 °C and maintained in ACSF at 25°C for at least 1h before use.

### Electrophysiological recording

The slices were submerged in the recording chamber and perfused with ACSF containing (in mM): NaCl (125.8), NaHCO3 (26), NaH2PO4 (1.2), KCl (3.1), CaCl2 (2.2), MgCl2 (1.4) and D-glucose (10) at a rate of 2-4 ml/min at 25 °C. AS86 or mIgG were perfused at a concentration of 15 nM. For LTP experiments, brain slices were perfused for at least 30 min prior to the induction of LTP.

All electrophysiological recordings were performed with an Axopatch-700B amplifier (Molecular Devices, USA) at the sampling rate of 10 kHz and filtered at 5 kHz using a Digidata 1550B analog-digital converter (Axon Instruments). Field EPSPs were recorded using 1.5-3.5 MΩ glass pipettes filled with ACSF and placed in the stratum radiatum of the CA1 region. Data were acquired and subsequently analyzed using ClampFit 10.4 software (Molecular Devices, USA). A bipolar stimulating electrode was positioned at the terminals of the Schaffer collateral (SC). Input/output relation of EPSP was assessed by electrical stimulation with intensities ranging from 100 μA to 1000 μA in 100 μA increments. Stimuli were delivered every 30 s and repeated for five times at each intensity. Paired-pulse responses were evoked at inter-stimulus intervals of 10, 20, 40, 60, 100, 150, 250 and 500 ms using a stimulation intensity of 0.5 mA. The paired-pulse ratio is defined as the ratio of second population spike amplitude to the first population spike amplitude. For LTP experiments, baseline responses were recorded for 15 min prior to stimulation. Electrical stimulation was delivered every 30 s in recordings, consisting of low-intensity, square-wave pulses (0.1 ms). LTP was induced by high frequency stimulation (1 s burst of equally spaced pulses at 100 Hz). Responses were subsequently recorded for an additional 60 min to monitor changes in synaptic transmission. The synaptic strength was determined by the slope from 10% to 90% of the rising phase of the field excitatory postsynaptic potential (fEPSP). The magnitude of LTP was quantified as the normalized average slope of the fEPSP taken from the last 10 min of recording.

### Congo red staining

Congo red staining was performed according to standard protocols. Frozen brain sections were prepared at the thickness of 30 μm and immersed in congo red solution (160 ml methanol, 40 ml glycerol mixed with 1 g Congo Red (Sigma, C6767)) for 15 min, and then dissolved in 200 ml 80% ethanol mixed with 0.4 g KOH for 1 min twice. After rinsing with distilled water, the slices were counterstained in hematoxylin (Solarbio life sciences, G1142), rinsed again, and dehydrated in ethanol and xylene. The bright field images were captured using ZEISS Axio Scan.Z1 (10 ×), and analyzed with Image J.

### Synaptophysin immunostaining

Immunohistochemistry with 30 µm frozen brain sections was performed as previously described 103. Images were captured with a Nikcon confocal microscope in 12 bit (1024 × 1024) using a 100 × Oil-immersion objective at a 3 × zoom. Each image was taken from CA1 of hippocampus and consisted of five steps of z stack pictures with a depth interval of 0.8 μm. The spine numbers were analyzed using Imaris software SPOT module with the same spot definition standard, and the spine numbers were calculated automatically.

### Behavioral tasks

The mice were handled more than 5 days (5 min for each mouse per day) before each behavioral task.

### Open field test (OFT)

The open field test was performed in a cubic open filed arena (made of white PVC). Mice were allowed to explore in this tank for 10 min in dark. The activity of mice was monitored by an infrared camera which is mounted above the arena and connected to an automated video tracking system and EthoVision XT software (Noldus, Netherlands). The arena was thoroughly cleaned with 75% alcohol after each trial.

### Analysis of cutaneous sensitivity with von Frey and Plantar test

One day after the tail vein injection of the TrkB antibody AS86, analyses of cutaneous sensitivity were performed with von Frey and Plantar hot plate tests. For the von Frey test, the up and down method and threshold calculation, first described by Chaplan et al., (1994) was used. The mice adapted on the mesh surface for at least 1 hour. Then, von Frey filaments were used to stimulate the bottom (midplantar) surface of the mice hind foot. Nine filaments represented 0.02, 0.04, 0.07, 0.16, 0.40, 0.6, 1.0, 1.4 or 2.0 g respectively. The first filament applied is a medium force (0.40 g). The positive response was described as rapid claw extraction, licking or shaking during stimulation or after removal of filaments. Here, we followed a modified protocol 104. For hot plate test, we used the instruments of Plantar Test (Ugo Basile, Comerio VA, Italy). The standard operation manual was followed. The strength of stimulation (IR value) was adjusted to produce baseline latencies of 8 to 10 seconds. Animals were first acclimatized in the apparatus for at least 1 hour before measurements. Each measurement was repeated at least 4 times with the intermission of 10 minutes.

### Novel object recognition test (NORT)

The novel object recognition test was conducted in cubic arena (33 cm × 33 cm × 20 cm made of white PVC). The experiments were performed in the dark. The mice were allowed to accommodate the empty arena for three consequent days (5 min/day). At the fourth day, the mice were allowed to explore two same adjacent objects (40 ml flasks filled of yellow sand) situated at 10 cm away from the arena wall for 5 min. After a 3h interval, one of the flasks was replaced by a new object (60 ml glass reagent bottle filled with blue sand), and the mice were allowed to explore for 5 min. The arena and the objects were thoroughly cleaned with 75% alcohol, and the alcohol was blown away by a fan after each trial. The numbers of nose pokes of the mice to each object were scored by blinded observers.

### Morris water maze (WM) task

The maze was made of a white inside circular tank (diameter = 120 cm) and was filled with white water at 21 ± 1 °C. The tank was equally divided into four quadrants (north, south, east, and west). An escape circular platform (diameter = 12 cm) covered with gauze for gripping was placed in the center of one quadrant and was hidden 1cm below water level. Four spatial cues were located on the inside walls of the circular tank, and the human tester always left the room in the same location during testing. For three rounds of testing, the platform was posited in a fixed position (WM1: SW, WM2: NE, WM3: SE) during the 4-5 days of learning trials. A camera located above the tank was interfaced with a computer, and EthoVision XT software (Noldus, Netherlands) was used to collect and analyze data. The mice were subjected to learning trials four times a day for 5-6 consecutive days, with a 25-30 min inter-trial interval. During each trial, the mice were released into water gently facing the tank wall from a principle start location as described previously 105, and were allowed to find the hidden platform for 60 s. They were to be put on the hidden platform manually for 10 s if the mice failed to find it within 60 s. On the probe day, a trial was carried out where the platform was removed, and the mice were allowed to swim for 60 s in the tank.

## Supplementary Material

Supplementary figures and tables.

## Figures and Tables

**Table 1:** Two-way ANOVA analysis for Primary latency in the probe test of water maze

	WM1			WM2			WM3		
Source of Variation	DF	F	P value	DF	F	P value	DF	F	P value
Interaction	1	3.834	0.0559	1	1.066	0.3077	1	0.06883	0.7944
Factor A (WT. vs. APP/PS1)	1	3.515	0.0668	1	8.276	0.0062 **	1	10.66	0.0022 **
Factor B (mIgG. vs. AS86)	1	0.2888	0.5934	1	4.68	0.0361 *	1	0.03029	0.8627
Residual (Error)	49			43			40		

**Table 2:** Two-way ANOVA analysis for Platform crossover in the probe test of water maze

	WM1			WM2			WM3		
Source of Variation	DF	F	P value	DF	F	P value	DF	F	P value
Interaction	1	11.63	0.0013 **	1	0.00598	0.9387	1	0.6443	0.427
Factor A (WT. vs. APP/PS1)	1	3.754	0.0586	1	17.51	0.0001 ***	1	7.811	0.008 **
Factor B (mIgG. vs. AS86)	1	1.185	0.2818	1	4.795	0.034 *	1	2.234	0.1431
Residual (Error)	48			43			39		

## Abbreviations

BDNF: brain-derived neurotrophic factor
TrkB: tropomyosin-related kinase B
AD: alzheimer's disease
Aβ: amyloid β
LTP: long-term potentiation
PI3K: phosphatidylinositol-3 kinase
Erk: extracellular regulated kinase
PLCγ: phospholipase C-gamma
p75^NTR^: p75 neurotrophin receptor
RTK: tyrosine receptor kinase
hTrkB: human TrkB
IgG: immunoglobulin G
EGR1: early growth response 1
NORT: novel object recognition test
WM: water maze
CNS: central nervous system
