# Supplementary material for: Therapeutic potential of a TrkB agonistic antibody for Alzheimer's disease
Source: Theranostics. 2020 May 23;10(15):6854–74. doi: 10.7150/thno.44165 (PMC7295064; doi:10.7150/thno.44165)
Supplement: Supplementary file 1 — Supplementary figures and tables. [file thnov10p6854s1.pdf]

Supplemental Material

Supplementary Figures

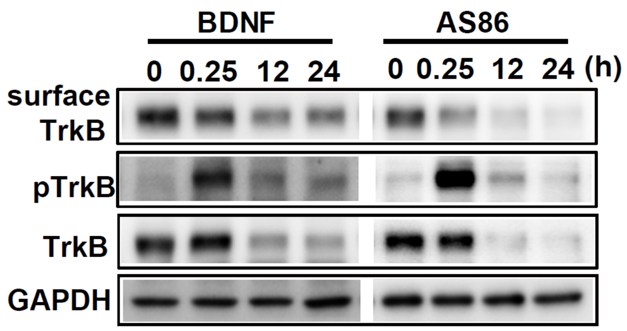

Figure S1. Endocytosis of TrkB induced by AS86. The changes of surface TrkB, pTrkB and total TrkB over time after BDNF or AS86 treatment are shown. Hippocampal neurons (DIV7) were treated with 3 nM BDNF or 10 nM AS86 for different amounts of time. Neuronal surface proteins were biotinylated for 30 min, lysed with RIPA buffer, and precipitated with ImmunoPure Immobilized Streptavidin. The precipitated cell surface TrkB, pTrkB and total TrkB were analyzed by Western blot analysis, using the indicated antibodies. The house-keeping protein GAPDH was used as a loading control. Note that similar to BDNF, AS86 induced a time-dependent endocytosis (decrease in cell surface TrkB) and intracellular degradation of TrkB.

A

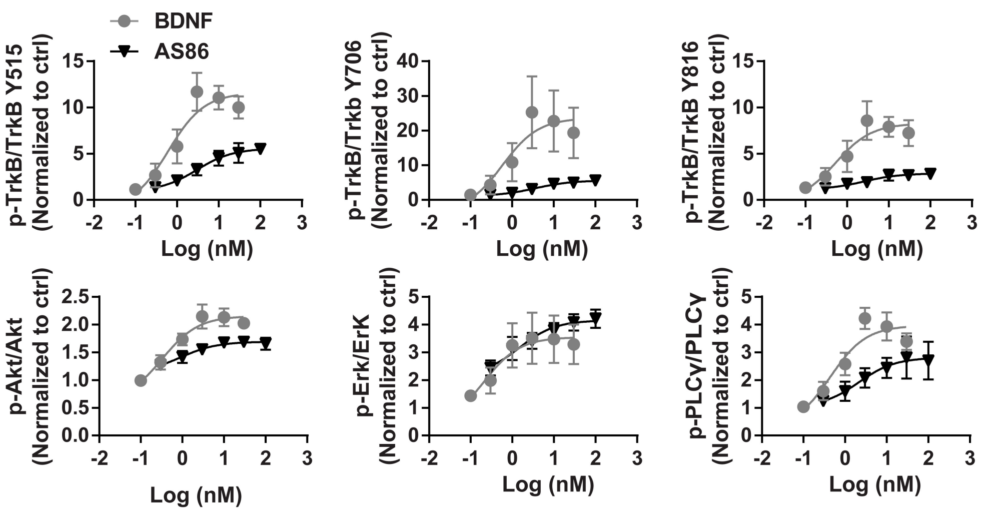

B

	BDNF EC50 (nM)	AS86 EC50 (nM)
pTrkB Y515	0.6066	2.959
pTrkB Y706	0.5845	3.535
pTrkB Y816	0.5213	2.542
pAkt	0.3297	0.9259
pErk	0.2031	1.460
pPLCγ	0.4350	2.134

C

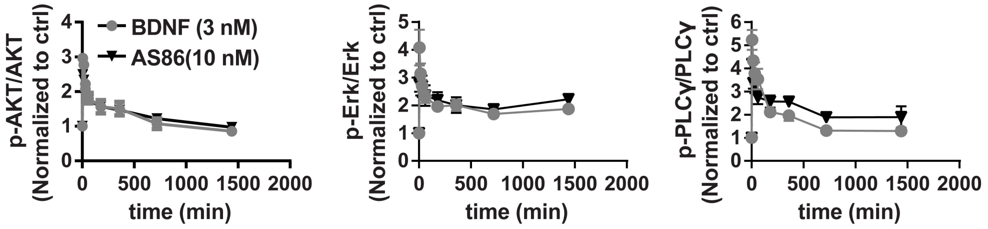

Figure S2. Potency and signaling of TrkB agonistic antibody AS86. (A-B) Dose response of TrkB activation and its downstream signaling in cultured hippocampal neurons. Primary hippocampal neurons (DIV10) were treated with different concentrations of AS86 or BDNF for 30min, and then the cell lysates were analyzed using Western blotting (N = 3 independent experiments, n = 3), as shown in Figure 1B. Quantifications for the ratios of phosphorylated protein to total protein were plotted as dose-response curves (A) and EC50s (B) were calculated. (C) Time course of AS86 downstream signaling in cultured hippocampal neuron. Primary hippocampal neurons (DIV10) were stimulated with AS86 or BDNF for different time, as shown in Figure 1C. The quantitative plots (C) are presented.

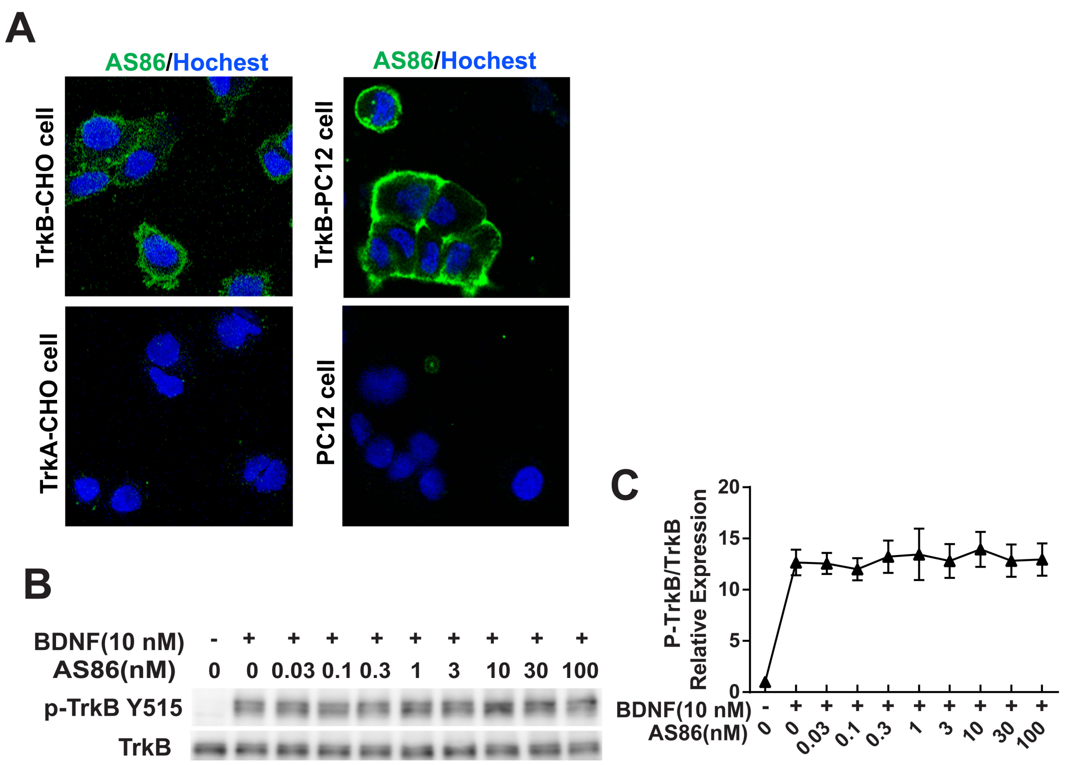

Figure S3. (A) Antibody specificity measured by immunostaining. TrkB-CHO, TrkA-CHO cells, TrkB-PC12 and normal PC12 cells were fixed and stained with the AS86 and normal IgG as the primary antibody. AS86 fluorescently labeled TrkB-CHO or TrkB-PC12 cells but not TrkA-CHO or normal PC12 cells. (B, C) Competition between AS86 and BDNF in TrkB activation. hTrkB-CHO cells were treated with a saturate BDNF (4nM) plus increasing concentrations of AS86 for 15 min. TrkB activity (pY515) were analyzed with Western blotting (n = 3). Representative Western blots (B) and quantitative plots (C) are presented.

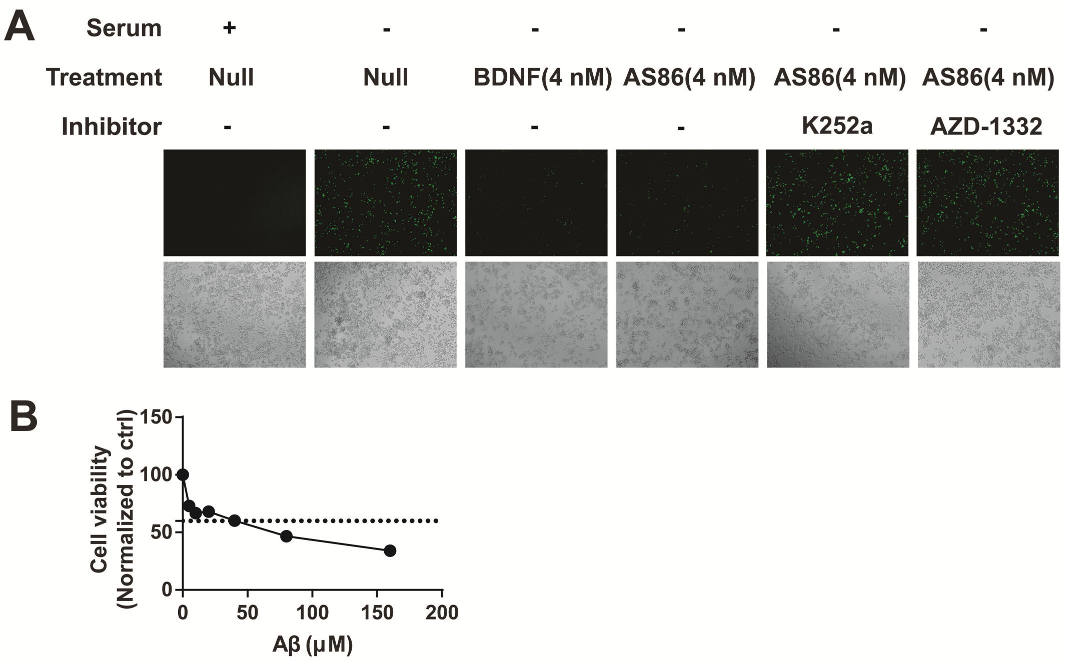

Figure S4. Attenuation of cell death by AS86. (A) AS86 rescued serum-deprivation induced cell death. PC12 cells in equal density were subjected to serum deprivation, followed by treatment of TrkB agonists at the present or absent of TrkB antagonists, as shown in Figure 3A. The representative fluorescent and phase contrast micrographs are presented. (B) Aβ-induced cell death. DIV8 hippocampal neuron were treated with different concentrations of Aβ (25-35) for two days and the cell viability levels were tested by ATP level quantification assay. A moderate concentration of Aβ (25-35) (5 μM) was selected to evaluate survival function of AS86. The dotted line represents 60% cell viability.

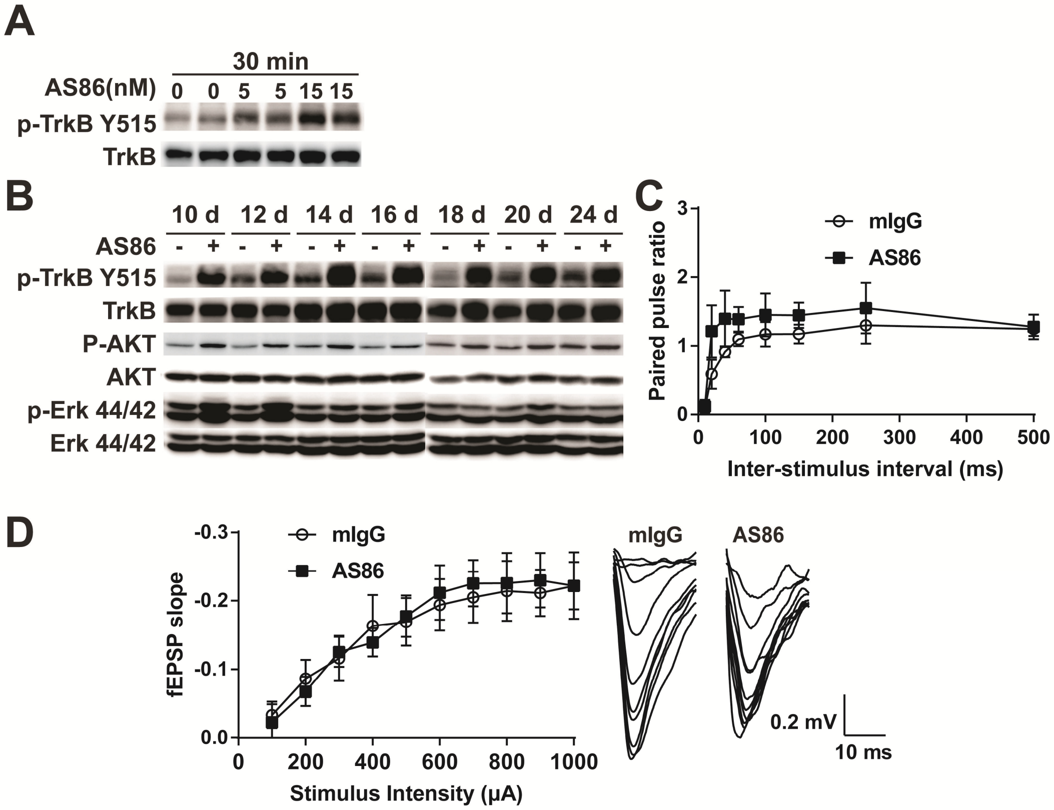

Figure S5. (A) Activation of TrkB by AS86 in hippocampal slices of different ages. Hippocampal slices (postnatal day 12, P12) were treated with AS86 (5 nM or 15 nM) for 30 min and the lysates were analyzed with Western blot. (B) Activation of TrkB and its downstream signaling pathways by AS86 in hippocampal slices derived from SD rats of different postnatal ages: P10, P12, P14, P16, P18, P20 and P24. The hippocampal slices were treated with 15 nM AS86 for 30min, and the lysates were analyzed by Western blotting using various antibodies as indicated. (C)Paired-pulse ratios (PPR) were evoked at different inter-stimulus intervals of 10, 20, 40, 60, 100, 150, 250 and 500 ms using a stimulation intensity of 0.5 mA (n = 6 slices). The bath application of AS86 did not change PPF at different time points. (D) Input/output curve was measured with different stimulus intensity. The right panels show the representative fEPSPs (n = 6 slices).

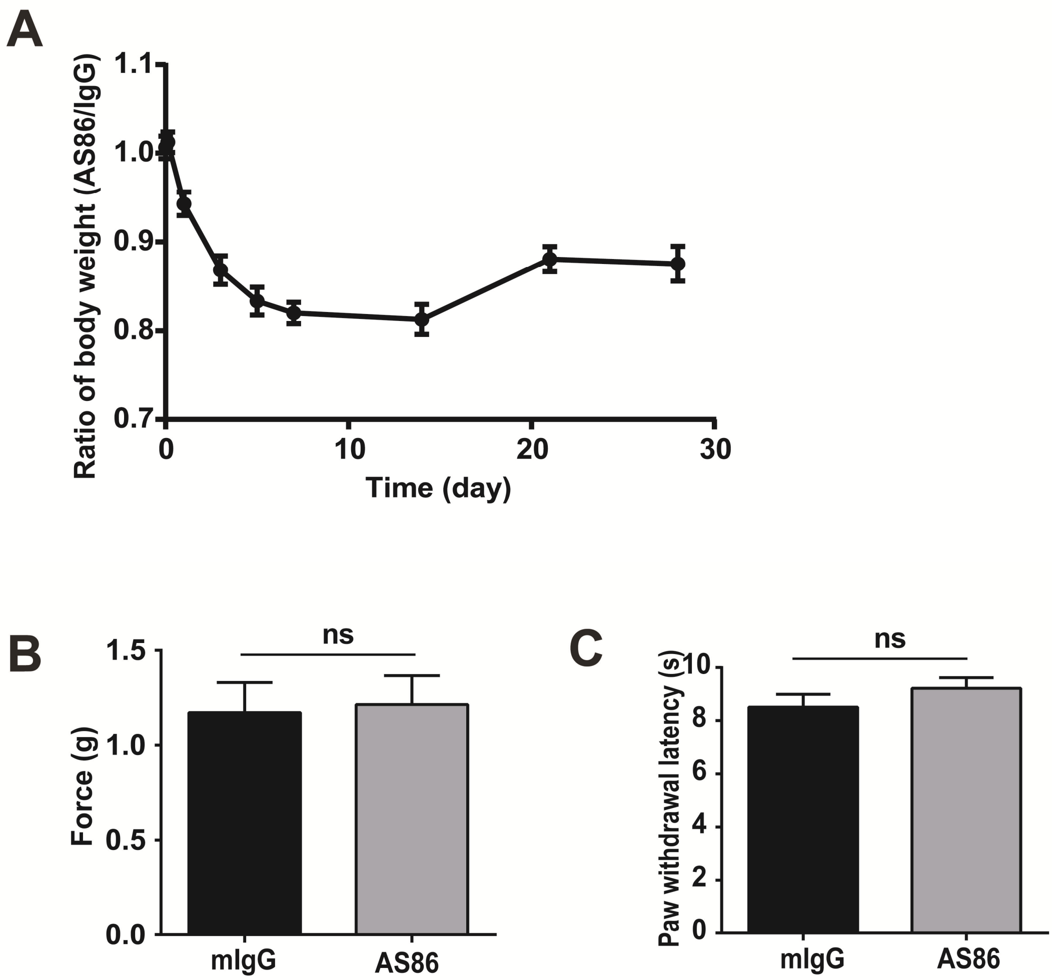

Figure S6. (A) Pharmacodynamic measurement of AS86. AS86 (1 mg/kg) or mIgG (1 mg/kg) were delivered to mice by tail vein injection, and body weights were measured at different time points (N = 10 mice). (B, C) Effect of AS86 on cutaneous sensitivity. Mice were treated with AS86 (2 mg/kg) or mIgG (2mg/kg) through tail vein injection. One day after the injection of the antibody, analyses of cutaneous sensitivity were performed by von Frey test (B) and Plantar hot plate test (C). Student's *t*-test. (N = 10 mice)

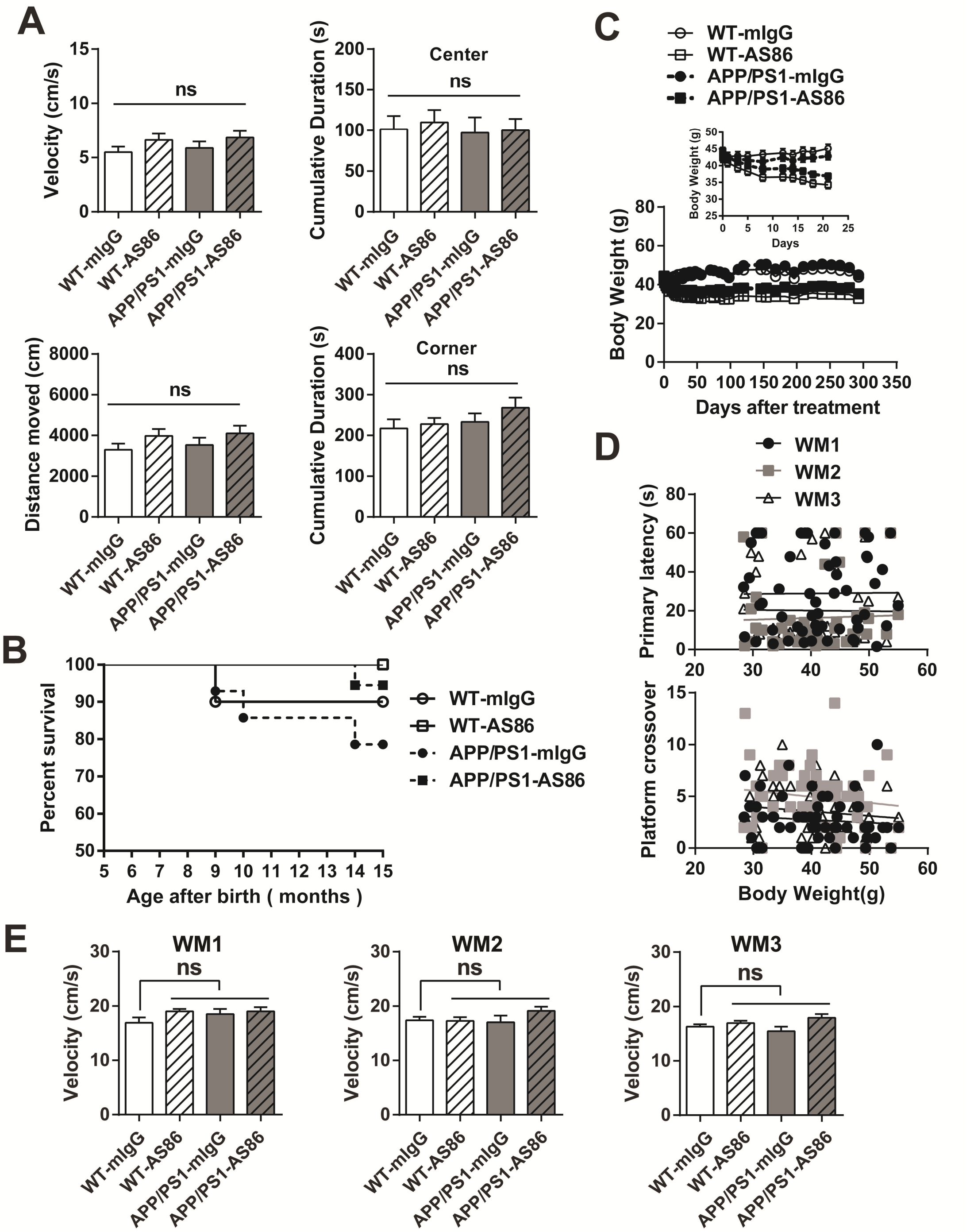

Figure S7. Assessment of behavioral and physiological effects of AS86. (A) Open field test (OFT). Mice (7 month old) were subjected to OFT after AS86 (1 mg/kg) treatment for 2 months. WT-mIgG (n = 7), WT-AS86 (n = 6), APP/PS1-mIgG (n = 7) and APP/PS1-AS86 (n = 6) mice were allowed to explore in the open field arena for 10 min. The velocity, moving distance, center duration and corner duration were quantified and presented in bar graphs. (B)Life span and (C) body weight of WT and APP/PS1 mice with different treatments. The upper panel is a magnified graph

showing that body weight changes in the beginning 20 days. Note that the body weights began to show differences after 3 days of AS86 treatment. (D) Assessment for the effect of body weight on WM test. The correlation of spatial memory with body weight for each WM test were plotted in the scatter diagrams. No correlation was found. (E) The swimming velocity of each mouse at the probe day was measured and quantified for each WM test.
